# The effects of step-count monitoring interventions on physical activity: systematic review and meta-analysis of community-based randomised controlled trials in adults

**DOI:** 10.1186/s12966-020-01020-8

**Published:** 2020-10-09

**Authors:** Umar A. R. Chaudhry, Charlotte Wahlich, Rebecca Fortescue, Derek G. Cook, Rachel Knightly, Tess Harris

**Affiliations:** Population Health Research Institute, St George’s, University of London, Cranmer Terrace, London, SW17 0RE UK

**Keywords:** Physical activity, Step-count monitoring, Pedometers, Smartphone applications, Fitness devices, Systematic review, Meta-analysis

## Abstract

**Background:**

Step-count monitors (pedometers, body-worn trackers and smartphone applications) can increase walking, helping to tackle physical inactivity. We aimed to assess the effect of step-count monitors on physical activity (PA) in randomised controlled trials (RCTs) amongst community-dwelling adults; including longer-term effects, differences between step-count monitors, and between intervention components.

**Methods:**

Systematic literature searches in seven databases identified RCTs in healthy adults, or those at risk of disease, published between January 2000–April 2020. Two reviewers independently selected studies, extracted data and assessed risk of bias. Outcome was mean differences (MD) with 95% confidence intervals (CI) in steps at follow-up between treatment and control groups. Our preferred outcome measure was from studies with follow-up steps adjusted for baseline steps (change studies); but we also included studies reporting follow-up differences only (end-point studies). Multivariate-meta-analysis used random-effect estimates at different time-points for change studies only. Meta-regression compared effects of different step-count monitors and intervention components amongst all studies at ≤4 months.

**Results:**

Of 12,491 records identified, 70 RCTs (at generally low risk of bias) were included, with 57 trials (16,355 participants) included in meta-analyses: 32 provided change from baseline data; 25 provided end-point only. Multivariate meta-analysis of the 32 change studies demonstrated step-counts favoured intervention groups: MD of 1126 steps/day 95%CI [787, 1466] at ≤4 months, 1050 steps/day [602, 1498] at 6 months, 464 steps/day [301, 626] at 1 year, 121 steps/day [− 64, 306] at 2 years and 434 steps/day [191, 676] at 3–4 years. Meta-regression of the 57 trials at ≤4 months demonstrated in mutually-adjusted analyses that: end-point were similar to change studies (+ 257 steps/day [− 417, 931]); body-worn trackers/smartphone applications were less effective than pedometers (− 834 steps/day [− 1542, − 126]); and interventions providing additional counselling/incentives were not better than those without (− 812 steps/day [− 1503, − 122]).

**Conclusions:**

Step-count monitoring leads to short and long-term step-count increases, with no evidence that either body-worn trackers/smartphone applications, or additional counselling/incentives offer further benefit over simpler pedometer-based interventions. Simple step-count monitoring interventions should be prioritised to address the public health physical inactivity challenge.

**Systematic review registration:**

PROSPERO number CRD42017075810.

## Introduction

Physical activity (PA) reduces all-cause mortality, and delivers important prevention and treatment benefits for many different physical and psychological conditions [1]. The World Health Organisation recognises physical inactivity as an important global health challenge, with more than a quarter of the world’s population not meeting current PA recommendations for health [1].

PA interventions can help tackle this global burden, with those employing behaviour change techniques being more effective [2]. Walking is the commonest PA and is an ideal behaviour for interventions to target as: both intensity and frequency can be gradually increased; it provides dynamic aerobic activity with minimal adverse effects [3]; and it can be a good way to achieve the PA guidance of 150 min of weekly moderate-to-vigorous PA [1, 4]. Different health drives to promote PA using walking, include campaigns to promote 10,000 steps per day [5], or adding in 3000 steps in 30 min to usual PA [6], with a recognition that for most people walking at a moderate intensity approximates to 1000 steps in 10 min [6].

Pedometers use body-worn motion sensors to objectively measure step-counts and are a simple, inexpensive intervention to increase PA levels [5]. Other monitors have gained popularity recently, due to technological advances and their ability to capture more data. These include accelerometers, measuring acceleration forces, incorporated into newer fitness technologies, such as body-tracking devices and smartphone applications, also providing objective PA measures, including step-counts [7]. Earlier systematic reviews and meta-analyses, focussing on pedometers, have highlighted significant increases in PA associated with their use [8, 9]. However, since these reviews, a number of larger randomized controlled trials (RCTs) with longer follow-up have been published [10–12]. Moreover, many trials incorporated within the earlier reviews included subjective self-report questionnaire outcomes, prone to particular biases [13] and with considerable over-reporting of PA compared to objective PA measures [14]. Finally, the effectiveness of newer fitness technologies, including body-worn tracking devices and smartphone applications in increasing PA levels needs to be explored systematically.

Thus, the main aim of this review was to examine the effects of step-count monitoring devices (pedometers, body-worn trackers and smartphone applications) on objectively measured step-counts among the adult general population.

Specific objectives were:

- To determine whether intervention effects varied with length of follow-up;
- To determine whether certain types of intervention were more effective than others (for example, studies reporting change in PA from baseline compared to end-point only, pedometers compared to other step-count monitors, and interventions providing additional counselling/incentives compared to those not).

## Methods

Our systematic review was performed and reported in accordance with the 2009 PRISMA statement [15].

### Search strategy

We searched for articles indexed in Ovid Medline, EMBASE, PsycINFO, Web of Science, Cochrane Library, CINAHL and ASSIA that were published from January 2000 using a combination of controlled vocabulary (for example, Medical Subject Headings (MeSH)) and keyword searching. RCTs published after the year 2000 were selected due to the emerging technology of measuring step-counts amongst the general population from this time onwards. The initial search was conducted in September 2017, with a forward citation search of included studies conducted in June 2018, and an updated search carried out in April 2020, to incorporate the most recent evidence and to ensure longer follow-up periods were included. Further studies were selected after examining the references of included studies and from earlier systematic review. Search strategies are provided for MEDLINE [Additional file 1] and other databases [Additional file 2].

### Study selection, inclusion and exclusion criteria

Study inclusion and exclusion criteria were developed in accordance with the PICOS framework [15, 16] and are fully described in Additional file 3. We included RCTs involving adults, published in English-language. Cluster randomised trials were eligible, as were cross-over studies, providing data were available at the end of the first phase to avoid possible intervention carry-over effects. Studies of healthy adults, those at risk of disease, or those with pre-existing chronic medical conditions (either physical or psychological) as expected within a general population were included. Studies were excluded if participants were hospital or institution-based, selected on the basis of a health condition; or involved high-performance training. Community-based programmes including step-count monitoring interventions (pedometers, other body-worn fitness devices and smartphone applications) and providing objectively measured step-count outcomes, either with change in step-count from baseline (change-on-change) or with only step-counts at follow-up (end-point only) were included. The intervention could feature any form of pedometer or any other device, either physical or electronic, which measured the user’s step-count, including mobile phone applications and common body-worn fitness devices. Outcomes reported at all time-points following intervention completion were included. Where studies had more than one step-count monitoring intervention, all were selected. Comparators included control group participants predominantly receiving ‘usual standard care’ or healthcare advice with minimal active engagement (that is, not using a step-count monitoring device). Where there was more than one control group, the one with least active engagement was selected. This review focussed on RCTs as these are considered the gold-standard for assessing interventions; randomisation, when correctly implemented, eliminates bias in treatment assignment and limits confounding. The search was restricted to English-language studies due to the lack of funding, as specified in our protocol.

References were initially managed using EndNote X7.7.1 [17], where duplicates were removed. We applied an RCT classifier [18] and studies deemed to have a ≤ 5% chance of being an RCT were excluded, after validation on a 5% sample. The RCT classifier works by utilising a machine learning algorithm to accurately identify randomised controlled trials [18]. This was possible for the initial search conducted in September 2017, but was not available for the later updated search conducted in April 2020. Two review authors then independently screened the remaining titles and abstracts using an inclusion check-list [Additional file 4] and Rayyan (a web application to rapidly screen a large number of records for selection) [19]. Full-texts of potentially eligible studies were obtained and assessed by two authors independently, with discrepancies resolved through discussion and consultation with the wider author team (RF / TH / DC).

### Data extraction

Data on characteristics of included studies were independently extracted by two reviewers (UC and RK) using a pre-piloted data extraction sheet. Data included: 1) study design, total participants, participant demographic details, follow-up length; 2) intervention details; 3) outcome measures. Where further clarification was required regarding data, study authors were contacted to provide this information. The collected data for the 32 studies which provided mean between-group difference in change from baseline step-count (change-on-change) and the 25 studies which provided end-point only step-counts are provided in Additional file 5.

### Risk of bias assessment

The risk of bias was assessed independently for each study by two reviewers independently, using the Cochrane Risk of Bias tool [16]. Disagreements were resolved through discussion with a third author (RF). A sensitivity analysis based on the Risk of Bias score was not performed, as this was not pre-specified in the protocol.

### Differences from published protocol

Compared to the outlined protocol, one of the secondary objectives focusing on different sub-groups based on demographics was not undertaken due to the lack of sufficient data in many studies. Nor did we analyse data on moderate-to-vigorous physical activity (MVPA) as a secondary outcome; this was problematic due to the heterogenous reporting of MVPA outcomes and was unnecessary because of the ubiquitous availability of step-count data.

### Data analysis

The primary outcome was step-count, reported with 95% confidence intervals (CI). We preferred change in step count from baseline as we anticipated within-person scores to be correlated and that change would result in greater precision and be less prone to bias, since individuals are used as their own controls, than studies based on measurements at follow-up only. If both change from baseline and end-point scores were available, we extracted both. If available, we preferentially used analysis of co-variance (ANCOVA) measures of change in our meta-analyses. For trials with multiple relevant intervention arms, we performed adjustments to the data before performing the meta-analysis, by splitting the comparator group to avoid double-counting. The I^2^ statistic was used to measure the heterogeneity [20].

Initial meta-analyses were performed and Forest plots created using RevMan 5.3 [21]. As we expected to find between-study heterogeneity, random-effects models were used for the meta-analysis. Potential publication bias was assessed using funnel plots, with sub-setting of the estimates by time of follow-up from randomisation.

We performed sub-group analyses for different follow-up periods and meta-regression (described in more detail below) to explore: 1) study outcome type (that is, change-on-change versus end-point studies); 2) type of monitor (that is, pedometer-based interventions versus other step-count monitoring interventions such as body-worn trackers and smartphone applications); and 3) intensity of intervention (that is, comparing interventions additionally offering individual / group counselling or financial incentives, with those that did not). We were unable to explore effect modification by demographic variables or to compare interventions with different contact lengths/ intensities due to the diversity and lack of reporting of these variables.

Additional meta-analyses were carried out using the Stata procedure Mvmeta [22, 23]. Specifically: (i) analysis of estimates changing over time was assessed using multivariate meta-analysis which formally allowed for the same studies appearing at different time points – the outcome was a matrix of 5 variables, with each column corresponding to one of the 5 time points and each row corresponding to a study. Studies contributed data at variable numbers of time points; a-priori we decided that this analysis should be restricted to studies providing change-on-change estimates as the correlation between different time points would differ in end-point only studies; (ii) multi-variable meta-regression was carried out at the first time point (≤4 months) to assess whether estimates of effect varied, as described above, by: study outcome type; type of monitor used; and intervention intensity. Details of the Mvmeta options used are given in Additional file 6.

Our review protocol was registered with PROPERO on the 31st August 2017 (registration number: CRD42017075810).

## Results

### Study selection

**Fig. 1:**
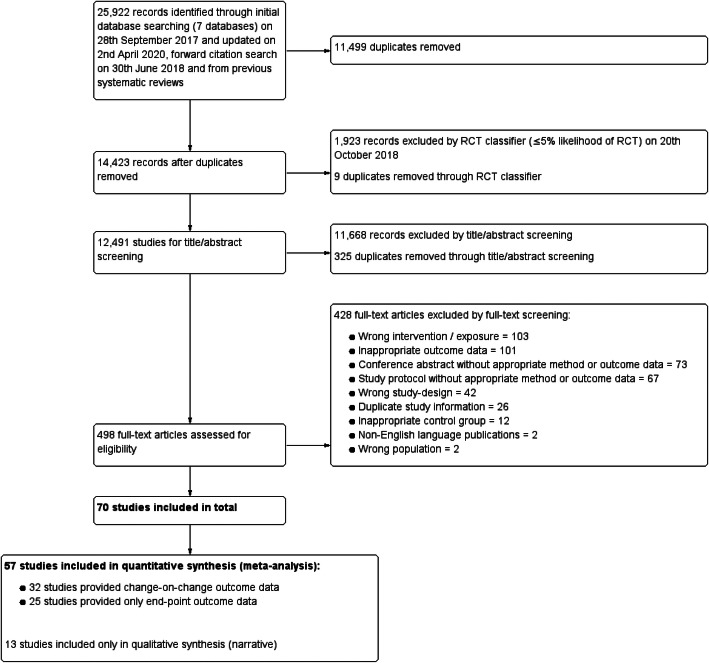
PRISMA Flowchart Screening of Literature Search and Eligibility

The PRISMA flow diagram for study selection can be seen in Fig. 1. 25,922 studies were initially identified using database searching in September 2017, forward citation search in June 2018, updated search in April 2020 and from previous systematic reviews, of which 14,423 records remained after removal of duplicates. After application of an RCT classifier 12,491 studies remained eligible for title/abstract screening, of which 498 full-texts were subsequently reviewed. 70 studies met the inclusion for the systematic review.

Therefore, 70 studies were included as part of this systematic review, of which:

- 32 studies provided data on mean between-group difference in change from baseline step-count (“change-on-change studies”) [10–12, 24–52];
- 25 studies only provided data on mean between-group difference in end-point step-count (“end-point only studies”) [53–77];
- 13 further studies did not provide data suitable for inclusion in our meta-analyses and were included as part of the narrative synthesis [78–90].

### Study characteristics

Full details of the 57 studies included in the meta-analyses and their study characteristics are shown in Additional file 5 [10–12, 24–77]. The remaining 13 studies are described later (see ‘narrative only studies’ for details) [78–90].

Of the 57 RCTs included in the meta-analyses, 54 RCTs had a parallel design [10–12, 24–41, 43–57, 59–72, 74–77]; 3 were cross-over studies [42, 58, 73]. Seventeen studies were conducted in the USA, 16 in the UK, 6 in Australia, 3 in Canada, Japan and Spain, 2 in Belgium, one each in Brazil, Hong Kong, Ireland, Netherlands, New Zealand, Singapore, and one was European-wide. One study identified with relevant step-count outcome data was a published abstract [64], with further details about the study highlighted in a separate paper [91]. The 57 RCTs included 16,355 participants with reported ages between 18 and 95 years, recruited from community-based, primary care settings, or from their workplace. The majority of participants in the studies providing ethnicity information were of white ethnicity. Participants were ambulatory with the ability to participate in step-count monitoring interventions. Most did not have specific risk factors, but included those at risk of developing Type 2 Diabetes Mellitus (T2DM), overweight with a body mass index (BMI) of ≥25 kg/m^2^, and those not meeting PA recommended guidelines. Thirty-nine [10–12, 24, 26, 33–39, 41, 42, 44, 48–50, 52, 54–60, 62, 64–70, 72–74, 76, 77] of the 57 RCTs used pedometers; the remaining 18 [25, 27–32, 40, 43, 45–47, 51, 53, 61, 63, 71, 75] used other step-count monitoring interventions such as other body-worn trackers and smartphone applications. The intensity of interventions ranged from the provision of a pedometer or other step-count monitor, often with PA electronic/print resources, written exercise programmes or on-line PA updates, to those that additionally provided individual PA consultations, group counselling or financial incentives [10–12, 24–77]. The control groups received usual care or electronic / print resources and educational sessions, but without the use of a step-count monitoring device. Thirty-two RCTs provided change-on-change step-count measures, 25 RCTs provided end-point only step-count measures. The length of follow-ups ranged from 1 week to 4 years.

### Risk of bias within studies

**Fig. 2:**
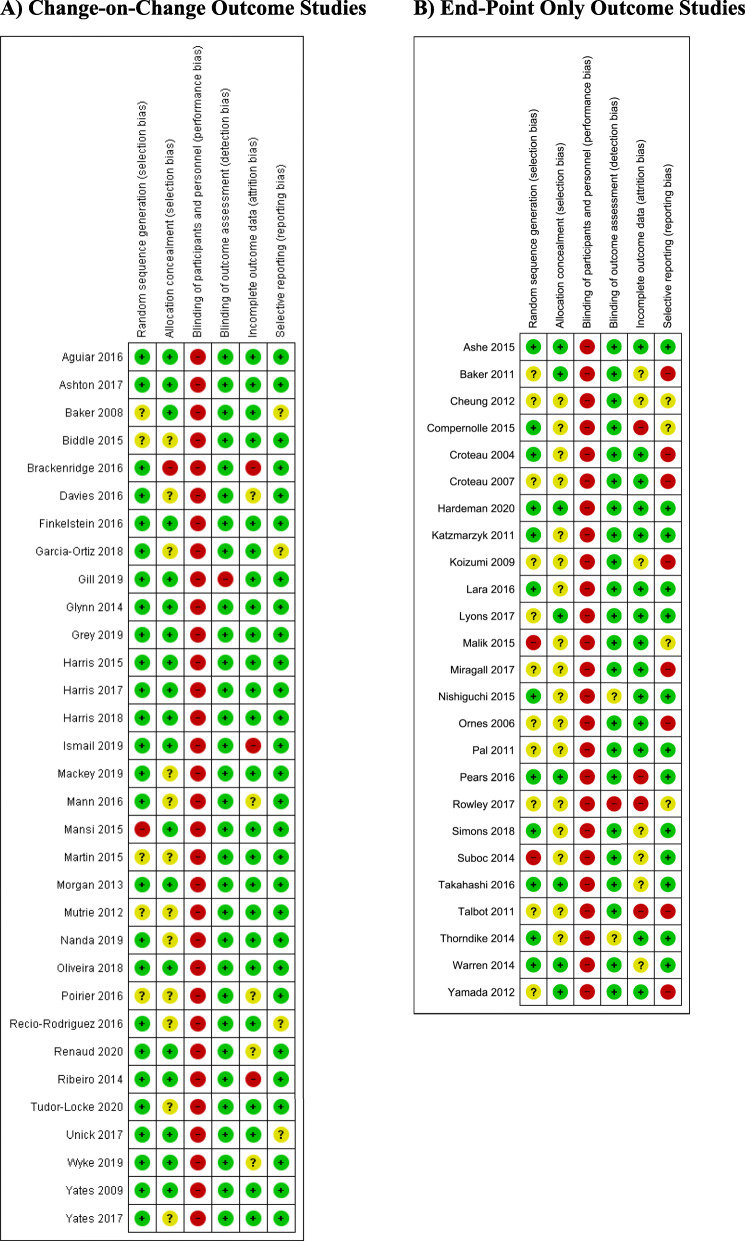
Risk of Bias Decisions for Change-on-Change and End-Point Only Outcome Studies. **a** Change-on-Change Outcome Studies. **b** End-Point Only Outcome Studies

The risk of bias analysis is shown in Fig. 2. Generally, the trials were reasonably well designed and conducted to have a low risk of bias, particularly those reporting change-on-change scores. This is despite the unavoidable risks associated with trials of behavioural interventions, which cannot be blinded and so all studies were judged to have a high risk of performance bias. Outcome assessment risk of bias (detection bias) was however generally rated as low, due to the objective outcome measures involved.

### Effects of interventions

#### Change-on-change studies

**Fig. 3:**
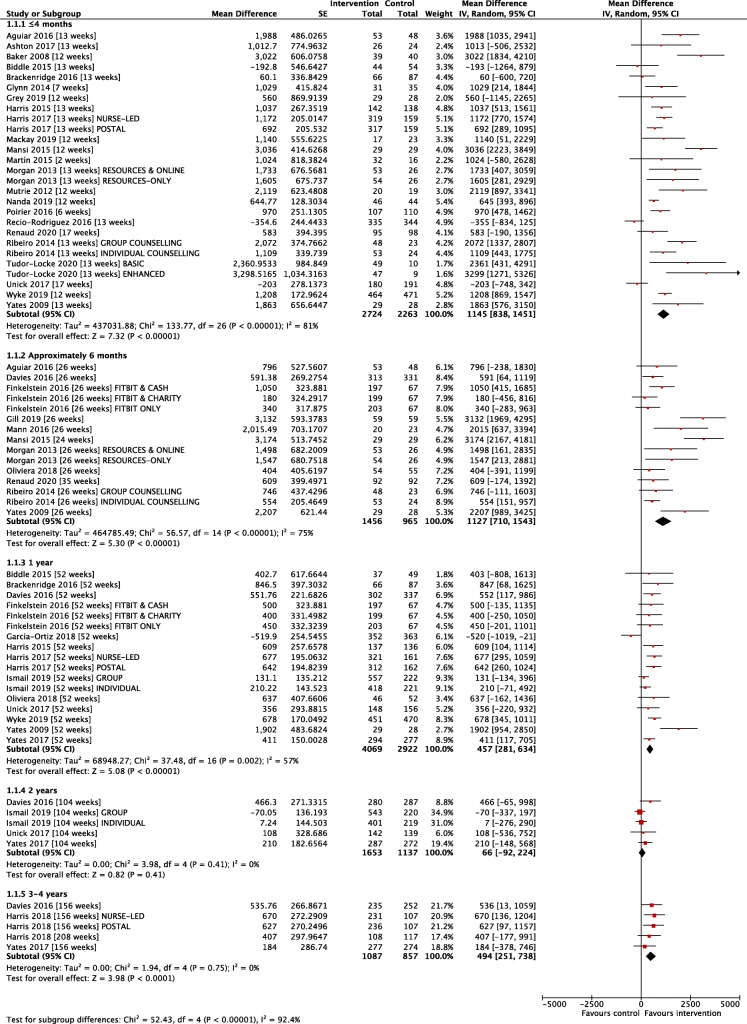
Mean between-group difference in change from baseline step-count (32 studies)

As planned, our primary analysis of variation in intervention effect during follow-up is based on the 32 change-on-change studies. Fig. 3 presents a forest plot of the individual and pooled effect estimates for the 32 studies, stratified by time of follow-up. At ≤4 months the pooled estimate of change indicated individuals in the intervention group were doing significantly more steps/day than controls: Mean difference (MD) + 1145 steps/day [95%CI: 838 to 1451; 23 studies; I^2^ = 81%]. A difference favouring the intervention group was maintained at 6 months MD + 1127 steps/day [95%CI: 710, 1543; 11 studies; I^2^ = 75%]. At 12 months the MD was + 457 steps/day [95%CI: 281, 634; 13 studies; I^2^ = 57%], at 2 years MD + 66 steps/day [95% CI: − 92, 224; 4 studies; I^2^ = 0%] and at 3–4 years MD + 494 steps/day [95%CI: 251, 738; 3 studies; I^2^ = 0%].

The multivariate results are very comparable to the univariate estimates (Table 1), at the shorter time periods (at ≤4 months MD + 1126 [CI: 787, 1466] and at approximately 6 months MD + 1050 [602, 1498]). The 1-year (MD + 464 [301, 626]) and 3–4 year estimates (MD + 434 [191, 676]) remain statistically significant in favour of the intervention group; however, the 2-year estimate (MD + 121 [− 64, 306]) based on four studies, one of which has a large weighting, is not statistically significant in favour of the intervention group.

**Table 1:** Analysis of Change-on-Change Studies at different Time-points: comparison of univariate analyses at each time-point with a multivariate analysis allowing for correlation of outcomes at different time-points

Follow-up Period	Separate Meta-analysis at Time-point Mean Difference [95% CI]	Multivariate Meta-analysis across Time-points Mean Difference [95% CI]
≤ 4 months	1172 [815, 1528]	1126 [787, 1466]
Approximately 6 months	1156 [672, 1641]	1050 [602, 1498]
1 year	458 [277, 638]	464 [301, 626]
2 years	66 [−92, 224]	121 [−64, 306]
3–4 years	494 [251, 738]	434 [191, 676]

Both Univariate and Multivariate models fitted using REML as MM option not available for multivariate model

#### End-point only studies

**Fig. 4:**
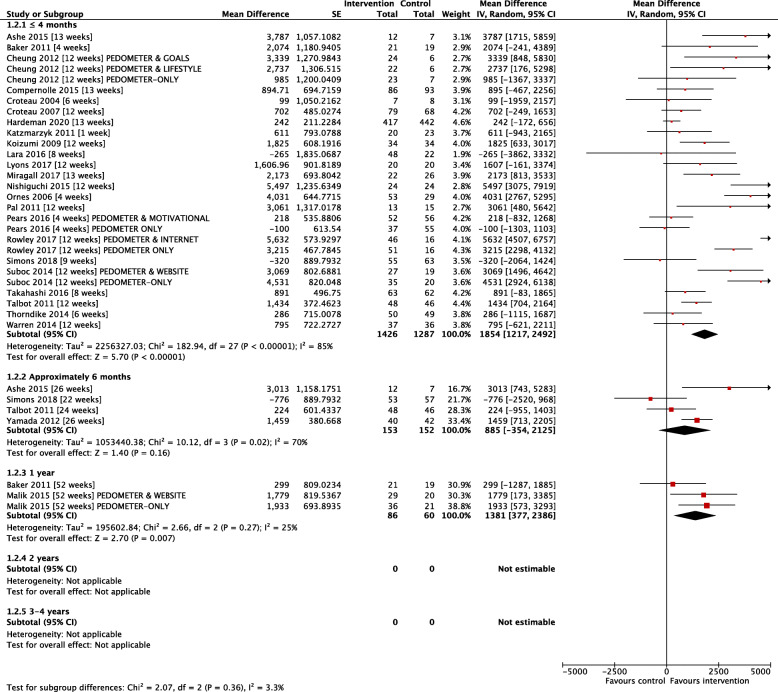
Mean between-group difference in end-point step-count (25 studies)

Figure 4 demonstrates the pooled effect estimates for the 25 end-point only studies at all reported time-points. At ≤4 months, the mean steps/day of the intervention group was significantly greater than control group: MD + 1854 steps/day [95% CI: 1217, 2492; 23 studies; I^2^ = 85%]. This was maintained at approximately 6 months with an MD + 885 steps/day [95% CI: − 354, 2125; 4 studies; I^2^ = 70%], albeit without reaching levels of significance, and at 12 months with a MD 1381 steps/day [95% CI: 377, 2386; 2 studies; I^2^ = 25%]. No follow-up data were available for end-point only studies beyond 1 year.

#### Analysis of outcomes at ≤4 months based on all study outcome type (change-on-change and end-point only studies)

**Fig. 5:**
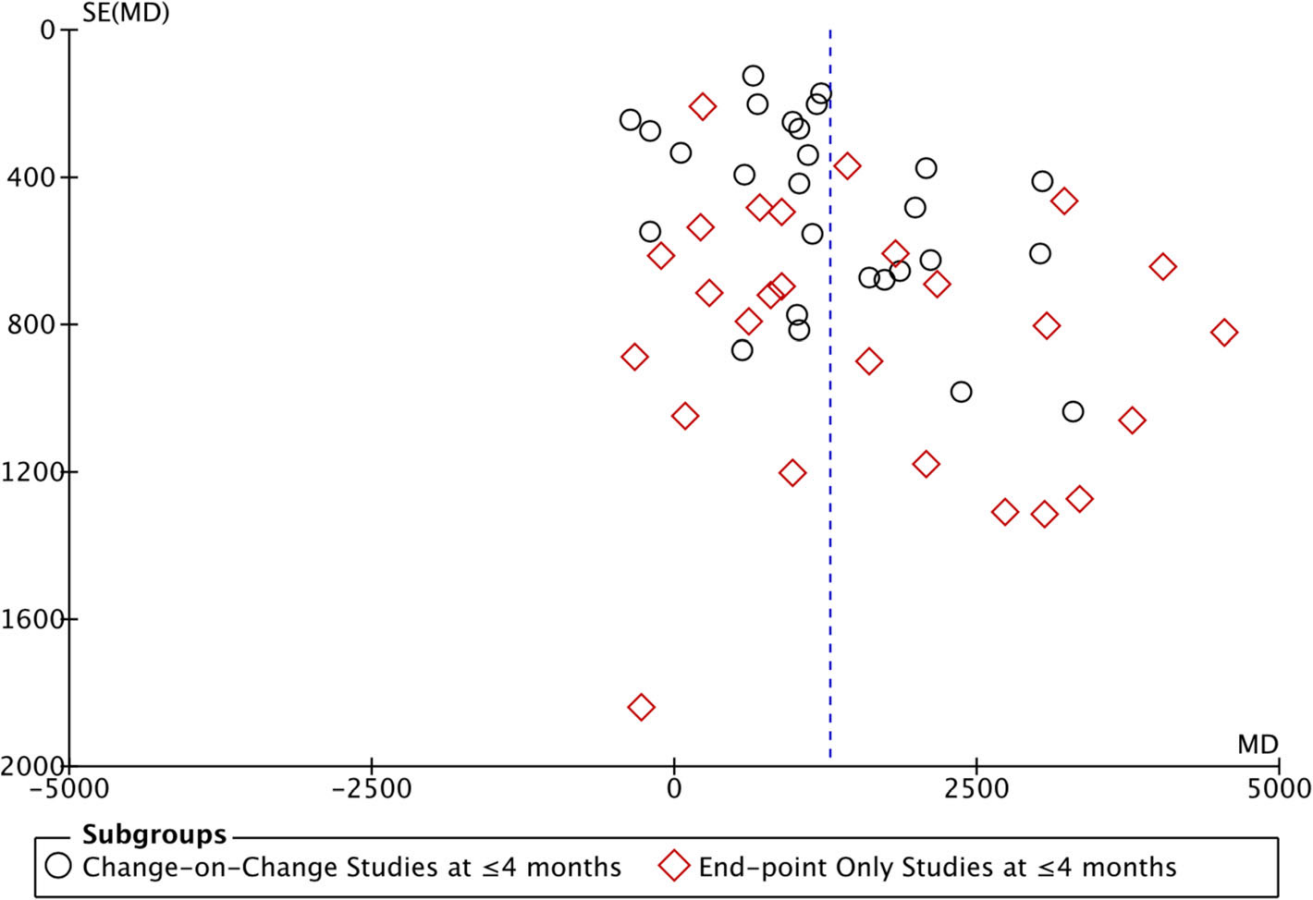
Funnel Plot of Studies reporting Mean-differences of Change-on-Change Study Outcome and End-point Only Study Outcome at ≤4 months Time-point

Figure 5 shows the funnel plot of change-on-change studies and end-point only studies at ≤4 months and demonstrates slight asymmetry.

Given the clear effect of time of follow-up on effect estimates and that the majority of studies reported effects at ≤4 months, we carried out meta-regression at ≤4 months’ time-frame only; the results are presented in Table 2. In keeping with the forest plot, end-point only studies reported bigger effect estimates than change-on-change studies, but this difference was not statistically significant: MD 616 steps [95%CI [− 92, 1324]. This effect was more than halved when mutually adjusted for ‘type of monitor’ and ‘intensity of intervention’: MD 257 [− 417, 931]. Studies using other step-count monitoring interventions (such as body-worn trackers and smartphone applications) reported lower treatment effects than those using pedometer-based interventions reported larger; MD − 927 steps [− 1633, − 190]; the effect was little affected by mutual adjustment for ‘study outcome type’ and ‘intensity of intervention’ (MD − 834 steps [− 1542, − 126]. Finally, studies which we rated as having higher intensity interventions (for example, those including additional PA individual or group counselling or financial incentives) had lower average effect estimates than those studies not using counselling or offering other incentives: MD -931 [− 1623, − 239], and this was slightly reduced by mutual adjustment for ‘study outcome type’ and ‘type of monitor’: MD -812 [− 1503, − 122]. A sensitivity analysis excluded 2 studies where the intervention was a smartphone only, with no body worn tracker [32, 46]; this made minimal difference to the estimate for other (body worn) step-count monitoring interventions, which still remained significantly lower than for pedometers.

**Table 2:** Univariate Meta-regression Analysis at First Time Point (≤4 months) to Investigate Effect-Modifiers

Effect Modifier	Factor	Univariate Mean Difference [95% CI]	Univariate Difference between Effect Modifier Groups Mean [95% CI]	Mutually Adjusted Difference between Effect Modifier Groups Mean [95% CI]
Study Outcome Type	Change-on-Change Studies	1215 [737, 1692]		
	End-point Only Studies	1831 [1307, 2355]	616 [−92, 1324]	257 [−417, 931]
Type of Monitor	Pedometer	1716 [1303, 2129]		
	Other Step-count Monitoring Interventions	789 [178, 1401]	−927 [−1633, −190]	−834 [−1542, −126]
Intensity of Intervention^a^	Intervention without PA individual or group counselling	2067 [1520, 2614]		
	Intervention with PA individual or group counselling or other incentives (e.g. cash or charity)	1136 [711, 1561]	−931 [−1623, −239]	−812 [−1503, −122]

^a^4 studies had missing grade

Above all based on REML models. Using mm2 model has little impact on meta-regression

### Narrative only studies

Thirteen studies were included in our systematic review, but were not included as part of the meta-analysis. Three studies provided insufficient overall intervention or control group data, and instead provided only sub-group analyses [78–80]; two of these studies concluded in favour of utilising step-count monitoring interventions to increase overall PA [78, 79], however the third study concluded that there was no evidence of efficacy [80]. A further multi-intervention arm study which provided data in the form of relative change scores also highlighted the use of a pedometer as a sufficient tool to increase steps [81]. Four studies were cross-over studies and did not provide data at the end of the first period [82–85]; there were inconsistent findings with studies reporting significant favourability towards utilising step-count monitoring interventions [82–84], and some reporting no significant differences between intervention and control groups [85]. The remaining five studies presented data graphically using either scatter graphs or bar charts, and all provided evidence to suggest that the intervention group incorporating step-count monitoring devices significantly increased the step-count at different follow-up periods [86–90], consistent with the findings from the meta-analyses.

## Discussion

### Principal findings

The use of community pedometer-based or other step-count monitoring interventions compared to usual care is associated with a significant increase in step-count when assessed using both change from baseline scores and end-point only scores. The greatest difference in steps is seen at the shortest follow-up period at ≤4 months, with intervention participants in change-on-change studies walking on average 1126 more steps/day. This significant improvement in step-count remains at approximately 6 months and 1 year with step-count monitoring interventions providing 1050 and 464 additional steps/day respectively. This overall improvement in steps is maintained at longer-term follow-up periods with differences of 121 steps/day at 2 years without reaching levels of significance and 434 steps/day at 3–4 years. Studies that presented outcome data using end-point only step-counts broadly supported these findings from change-on-change studies, but provided no data beyond 1 year. Our meta-regression analyses strongly suggest that newer devices such as body-worn trackers and smartphone applications are less advantageous than simpler pedometers, while interventions including individual or group counselling, or financial incentives, also offered no additional step-count benefit compared to those interventions without. Taken together these findings suggest that simple pedometer-based interventions can lead to both short and long-term PA increases and should be considered more widely for public health PA promotion.

### Study strengths and weaknesses

There are several strengths to this systematic review and meta-analysis. We followed PRISMA guidance and prospectively published our protocol. We were able to incorporate recent larger trials that had not been previously included in systematic reviews. Our review incorporated data from over 16,000 participants from 70 eligible studies (57 studies included in meta-analyses) to provide robust conclusions about the effect of step-count monitoring interventions. We also incorporated different methods of measuring our primary outcome, using both change from baseline and end-point only daily step-count measurements, allowing us to combine more studies and to further strengthen our conclusions. Given that the vast majority of studies had an intervention period of approximately 3 months, and the availability of data at 1 year and beyond, our analyses provide clear evidence of maintenance of an effect many months after the intervention has finished. The risk of bias assessment demonstrated that most studies, particularly those reporting change-on-change scores, used appropriate methods to minimise bias, despite the challenges with blinding such interventions. An updated search carried out in April 2020, ensures that this review captures the most recent data relevant for this systematic review. To our knowledge, this is the first review to directly compare the effectiveness of body worn devices and smartphone applications with traditional pedometers in general population samples.

There are also limitations to consider. Since all studies had unblinded participants, the risk of performance bias was judged high, although this is a universal issue with such interventions. Moreover, we were confident that due to the objective outcome measures the risk of detection bias was low. Further evidence to support this comes from trials that have increased step-counts in the long term and have also reduced important clinical outcomes such as fractures and cardiovascular events [92]. Many studies did not mention the theoretical underpinning underlying their behavioural change interventions, and as such we felt that it would be difficult to compare the effect of different studies on this basis. We also explored the potential of publication bias using a funnel plot; the slight asymmetry, particularly amongst the end-point only studies, indicated that smaller, shorter trials with positive results may have been preferentially published. We feel however, that this would have had only a small impact on our overall findings and it confirmed our preference for change-on-change step-count data as our main analysis. We recognize that there was a high degree of statistical heterogeneity, especially at the short follow-up periods, probably due to the considerably varied nature of the study designs and different intervention types.

At the 2-year time-point, the estimated treatment effect is not statistically significant; this is mainly due to the Ismail et al study, which has a weighting of 65.9% within this follow-up period [36]. This study was not effective at increasing step-counts at either the 1 year or 2-year follow-up points, for which the authors offer several explanations [36]. There was a lack of engagement with the intervention with a delay between randomisation and intervention commencement [36]. Of participants randomised to the two interventions, 28% did not start the intervention, while an additional 17% did not complete it [36]. Loss to follow-up at 24 months was also significantly higher in the intervention groups than the usual care group [36]. The authors speculated that study fatigue might explain this lack of engagement; interventions involved 10 sessions as well as considerable data collection at baseline 12 and 24 months [36]. This analysis of an unsuccessful complex intervention further strengthens our key conclusion that simple step-count monitoring interventions need to be prioritised.

An important limitation of all meta-regressions is that they are essentially observational comparisons of different trials and lack the formal rigour of within trial randomised comparisons. In the future, trials may compare different types of interventions by randomising within trial; any such trials will be better informed by the between trial comparisons we have presented. Unfortunately, many studies provided incomplete information about some aspects of the interventions such as the number and lengths of contacts, so this could not be formally analysed. Our analysis of intensity of intervention only included whether individual or group counselling or other incentives were offered, not the number nor length of contacts. We did not seek to obtain individual participant data and so were unable to explore other potential within trial effect modifiers, especially those related to demographic variables such as age, gender and socio-economic status. Finally, the studies were mostly conducted in high income countries and recruited participants of white ethnicity, so the results may not be as applicable to other countries and other ethnic groups.

### Comparison with other studies

Two previous systematic reviews focussed solely on pedometer-based interventions [8, 9]. Kang et al combined observational and randomised studies in both adults and children and reported a short-term increase of approximately 2000 steps/day [8]. Bravata et al demonstrated an improvement of 2491 steps/day in RCTs in adults; however, the trials included were small (maximum of 51 subjects randomised) and half were based on subjects with serious chronic diseases, which may not be generalizable [9]. Apart from inclusion of observational studies and a focus on specific diseases, other reasons that their estimates of effect were greater than our own, may include self-reported PA measures in some of their trials and small sample sizes [8, 9]. Our more conservative estimate of change in step-counts is potentially less biased, due to inclusion of larger RCTs, with longer follow-up periods and only using objectively measured outcomes. Other reviews included pedometer-based and other broader physical activity interventions (though not body-worn trackers or smartphone applications) with both objective and self-reported PA measures [93–95]. Hobbs et al found no relationship between intervention effectiveness and the number of intervention contacts or mode of delivery and found only limited PA data beyond 12 months [95]. Our review therefore builds on previous work on step-count monitors, by focusing solely on objective physical activity measures in the adult general population, and including a number of recently published larger trials with longer follow-up periods [10–12] and trials of body-worn fitness trackers [25, 27–29, 40, 43, 45, 47, 51, 53, 61, 63, 71, 75] or smartphone only applications [30–32, 46] or combined [25, 28, 40, 47, 53, 63, 71].

A very recent meta-analysis focussing on pedometer-based and accelerometer-based PA interventions amongst adults with cardio-metabolic conditions demonstrated small to medium PA level improvements [96]. Although this review had few long-term follow-up trials, it highlighted that pedometer-based interventions had improved association with PA as compared to accelerometer-based interventions [96], which is consistent with our findings comparing pedometer interventions with other body-worn monitors and smartphone applications. However, it demonstrated that the greatest increases in PA levels was achieved by more complex interventions, with regular contact with healthcare professionals, which differs from our own meta-regression analysis [96]. This may in part be due to our different definitions of complexity as well as to the different study populations; their study focused on adults with cardio-metabolic conditions, whilst ours focussed on the general population. It is worth emphasising that only 2 studies were included in both reviews [12, 60]. A further recent review of consumer-based wearable activity trackers (not pedometers), also demonstrated increases in steps of 500–600/day compared to controls, but did not provide any comparison with pedometer interventions and lacked long term data [97].

### Implications for public health research, practice and policy

From a research perspective, our review highlights two problems of inconsistent outcome reporting amongst RCTs. Some studies provided only graphical presentation of step-count results, which prevented their inclusion in meta-analyses. We were also unable to analyse the importance of both number and length of intervention sessions, due to reporting inconsistencies, which others have also highlighted [96]. Moving forward, therefore, when interventions are being investigated, it is important for study reports to provide clear information about the exact nature of the intervention, the number and length of contacts included within it and the reporting of outcomes, to provide further robust evidence to inform policy.

This review demonstrated that step-count monitoring interventions can lead to sustained increases in people’s walking, but that fitness trackers and smartphone application offered no clear advantage over simpler pedometer-based interventions. It is important to consider why this might be. Step-count monitoring interventions which have been shown to be particularly effective in long-term PA change, encompass behavioural change techniques, including goal setting, self-monitoring and feedback [98]. Pedometers can effectively provide this simple self-monitoring information with an easily understandable output [5, 98]. Fitness trackers also incorporate distance walked, elevation, the physical activity intensity, heart rate, rewards and social participation, amongst other features and have potentially been shown to be superior to pedometers [98]. However, when focussing on walking and increasing step-counts, small and simple goals may be more effective for long-term engagement of larger goals [98]. The ‘3,000 steps in 30 minutes’ is becoming an ever-increasing public health initiative [6], which is easily accomplished using a pedometer; more complex information is not required and may in fact detract from the simple message. In addition, provision of additional individual or group counselling or financial incentives also did not provide further benefit over simple step-count monitoring interventions, suggesting that a ‘less is more’ approach might be more suitable for walking interventions in community-based populations. This also has cost-effectiveness advantages [99], with positive implications for those commissioning PA interventions and services.

From a public health policy and practice perspective what does a long-term improvement of 434 steps per day at 3–4 years mean? Clear dose-response associations have been reported between increases in step-counts and reduced mortality, with 1000 extra steps/day being associated with a 15% lower risk in older men [100] and a 6% reduction in younger cohorts of men and women [101]. A recent meta-analysis also showed a clear dose-response association between accelerometer measured PA and all-cause mortality, with clear evidence that the strongest benefits were seen at lower PA levels, for all activity intensities [102]. Therefore, there is evidence to suggest that any form of activity, either moderate or lighter levels are still associated with improved mortality benefit [102–104], and this is reflected within recent PA guidance [4]. This has been further corroborated by a recent large population-based cohort study, which has highlighted that engaging in leisure time aerobic activity and meeting national PA guidelines reduces all-cause and cause-specific mortality [105]. Observational studies such as those above [100–105] are problematic however, in terms of potential reverse causality; so findings from two recent pedometer-based RCTs that demonstrated significant effects on clinical outcomes, with reductions in both cardiovascular events and fractures for an approximate increase of 400–600 steps per day 3–4 years post-intervention, provide reassuring support for these findings [92].

## Conclusions

Physical inactivity is an important public health concern and our findings strengthen the evidence that step-count monitoring interventions can improve long-term PA levels in adults. There was no clear advantage of newer body-worn trackers or smartphone applications over simple pedometers, and no evidence that additional individual or group counselling improved outcomes. These findings have important implications for those wishing to use PA interventions to address the public health inactivity challenge and suggest that simple pedometer-based walking interventions should be prioritised.

## Supplementary information

**Additional file 1.** MEDLINE Search Strategy.

**Additional file 2.** Outline of Search Strategy for Electronic Databases.

**Additional file 3.** Study inclusion and exclusion criteria using the PICOS tool.

**Additional file 4.** Title/Abstract Screening Reviewer Checklist Eligibility.

**Additional file 5.** Summary of Change-on-Change Studies (32 Studies) and End-point Only Studies (25 Studies) Characteristics.

**Additional file 6.** Stata procedure Mvmeta.

## Data Availability

All data generated or analysed during this study are included in this published article [and its supplementary information files]. There was no patient and public involvement in this study. Dissemination to study participants is not applicable. Data is shared in our supplementary material and will be on the St George’s, University of London data repository (URL will be provided at time of publication).

## Abbreviations

CI: Confidence Intervals
CW: Charlotte Wahlich
DC: Derek G Cook
MD: Mean difference
MVPA: Moderate-to-vigorous physical activity
PA: Physical activity
RCT: Randomised controlled trials
RF: Rebecca Fortescue
RK: Rachel Knightly
TH: Tess Harris
UC: Umar Chaudhry
