# Supplementary material for: The effects of step-count monitoring interventions on physical activity: systematic review and meta-analysis of community-based randomised controlled trials in adults
Source: Int J Behav Nutr Phys Act. 2020 Oct 9;17:129. doi: 10.1186/s12966-020-01020-8 (PMC7545847; doi:10.1186/s12966-020-01020-8)
Supplement: Supplementary file 1 — Additional file 1. MEDLINE Search Strategy. [file 12966_2020_1020_MOESM1_ESM.docx]

**Additional File 1: MEDLINE Search Strategy**

Ovid MEDLINE(R) Epub Ahead of Print, In-Process & Other Non-Indexed Citations, Ovid MEDLINE(R) Daily, Ovid MEDLINE and Versions(R)

RCT search adapted from Cochrane’s handbook: MEDLINE sensitivity maximizing strategy

1 Physical Activity/ (90858)

2 physical$ activ$.ti,ab. (92861)

3 Motor Activity/ (95692)

4 motor$ activit$.ti,ab. (15364)

5 Exercise/ (90858)

6 exercis$.ti,ab. (261700)

7 Physical Fitness/ (26504)

8 physical$ fit$.ti,ab. (8434)

9 Physical Exertion/ (59316)

10 physical$ exert$.ti,ab. (2105)

11 Walking/ (28465)

12 walk$.ti,ab. (100272)

13 1 or 2 or 3 or 4 or 5 or 6 or 7 or 8 or 9 or 10 or 11 or 12 (552401)

14 randomized controlled trial.pt. (482652)

15 controlled clinical trial.pt. (96900)

16 randomized.ti,ab. (454561)

17 placebo.ti,ab. (202095)

18 Clinical Trials as Topic/ (190590)

19 randomly.ti,ab. (293285)

20 trial.ti. (190212)

21 14 or 15 or 16 or 17 or 18 or 19 or 20 (1188803)

22 exp animals/ not humans.sh. (4589760)

23 21 not 22 (1096675)

24 pedomet$.ti,ab. (2340)

25 acceleromet$.ti,ab. (12149)

26 (step$ adj3 count$).ti,ab. (1894)

27 (step$ adj3 (day$ or daily or week$)).ti,ab. (3458)

28 activ$ monitor$.ti,ab. (3863)

29 (electronic$ adj3 (track$ or devic$)).ti,ab. (9264)

30 wearabl$.ti,ab. (5684)

31 Fitbit$.ti,ab. (218)

32 SenseWear$.ti,ab. (401)

33 Jawbone$.ti,ab. (764)

34 Fuelband$.ti,ab. (18)

35 Health$ Tracker$.ti,ab. (10)

36 DirectLife$.ti,ab. (11)

37 Vivofit$.ti,ab. (17)

38 Misfit$.ti,ab. (1540)

39 Polar$ Loop$.ti,ab. (38)

40 (mobil$ adj3 applicat$).ti,ab. (2358)

41 phon$.ti,ab. (59905)

42 apple$.ti,ab. (13742)

43 android.ti,ab. (1631)

44 24 or 25 or 26 or 27 or 28 or 29 or 30 or 31 or 32 or 33 or 34 or 35 or 36 or 37 or 38 or 39 or 40 or 41 or 42 or 43 (112180)

45 13 and 23 and 44 (3065)

46 limit 45 to yr="2000 -Current" (2960)

47 Infant/ (754480)

48 infant$.ti,ab. (371263)

49 Child/ (1599833)

50 child$.ti,ab. (1250127)

51 47 or 48 or 49 or 50 (2490169)

52 46 not 51 (2347)
