# Supplementary material for: The effects of step-count monitoring interventions on physical activity: systematic review and meta-analysis of community-based randomised controlled trials in adults
Source: Int J Behav Nutr Phys Act. 2020 Oct 9;17:129. doi: 10.1186/s12966-020-01020-8 (PMC7545847; doi:10.1186/s12966-020-01020-8)
Supplement: Supplementary file 2 — Additional file 2. Outline of Search Strategy for Electronic Databases. [file 12966_2020_1020_MOESM2_ESM.docx]

**Additional File 2: Outline of Search Strategy for Electronic Databases**

|  | Types of Study | Population | Intervention | Outcomes | Others |
| --- | --- | --- | --- | --- | --- |
| Definitions | RCTs | General Population  Aged >18 years  Generally healthy (medical conditions not focussed) | Measure physical activity  Pedometer and other step-count monitoring interventions, including: 1) pedometers; 2) handheld devices; 3) mobile phone applications  Community-based | Primary Outcome(s): change in step-count at follow-up compared to baseline  Secondary Outcome(s): distance travelled or time spent walking, or relevant accelerometer measurements e.g. time spent MVPA. Considering all follow-up periods: immediate, short-term, medium-term or long-term | Published after 1^st^ January 2000  Human studies |
| MeSH Terms | randomized controlled trial.pt.  controlled clinical trial.pt.  Clinical Trials as Topic/ | Infant/  Child/ | Physical Activity/  Motor Activity/  Exercise/  Physical Fitness/  Physical Exertion/  Walking/ () |  | exp animals/ not humans.sh.  limit to English language and yr="2000 -Current” |
| Keywords (ti,ab) | randomized.ti,ab.  placebo.ti,ab.  randomly.ti,ab.  trial.ti. | infant$.ti,ab.  child$.ti,ab. | physical$ activ$.ti,ab.  motor$ activit$.ti,ab.  exercis$.ti,ab.  fit$.ti,ab.  physical$ exert$.ti,ab.  walk$.ti,ab. | pedomet$.ti,ab.  acceleromet$.ti,ab.  ***Iterations of measurement:***  (step$ adj3 count$).ti,ab.  (step$ adj3 (day$ or daily or week$)).ti,ab.  activ$ monitor$.ti,ab.  (electronic$ adj3 (track$ or devic$)).ti,ab.  ***Wearables Brands:***  wearabl$.ti,ab.  Fitbit$.ti,ab.  SenseWear$.ti,ab.  Jawbone$.ti,ab.  Fuelband$.ti,ab.  Health$ Tracker$.ti,ab.  DirectLife$.ti,ab.  Vivofit$.ti,ab.  Misfit$.ti,ab.  Polar Loop$.ti,ab.  ***Mobile Phone Applications:***  (mobil$ adj3 applicat$).ti,ab.  phon$.ti,ab.  apple$.ti,ab.  android.ti,ab. |  |
