# Supplementary material for: The effects of step-count monitoring interventions on physical activity: systematic review and meta-analysis of community-based randomised controlled trials in adults
Source: Int J Behav Nutr Phys Act. 2020 Oct 9;17:129. doi: 10.1186/s12966-020-01020-8 (PMC7545847; doi:10.1186/s12966-020-01020-8)
Supplement: Supplementary file 3 — Additional file 3. Study inclusion and exclusion criteria using the PICOS tool. [file 12966_2020_1020_MOESM3_ESM.docx]

**Additional File 3: Study inclusion and exclusion criteria using the PICOS tool**

|  |  | **Inclusion Criteria** | **Exclusion Criteria** |
| --- | --- | --- | --- |
| P | Participant | - Aged 18 or over. - Healthy participants, or those who may be at risk of disease, or who do not have any medical conditions/risk factors that prevent them from undertaking physical exertion. These may include hypertension, hyperlipidaemia, dyslipidaemia, impaired glucose tolerance, overweight/obese, family history of medical conditions, metabolic syndrome or osteopenia. - Where appropriate, studies included participants who have been selected on the basis of their: a) demographics (including age and gender); b) level of pre-intervention physical activity; or c) pre-existing chronic medical conditions as highlighted above (either physical or psychological) as expected within a general population who are not necessarily the focus of the exposure. | - Children or adolescents under the age of 18. - Disease-specific studies or those selected on the basis of a health condition, for instance those studies that sub-categorise and focus on particular conditions including diabetes, chronic obstructive pulmonary disease (COPD), heart failure and osteoporosis. - Patients who were institutionalised in, for example. nursing homes, or residential care homes, or who are due to be admitted to hospital. - Those involved with high-performance training, for example, marathon runners. - Hospital-based or secondary care patients. |
| I | Intervention | - Measures of physical activity were an outcome component of the study, and obtained using objective techniques. - Feature any form of pedometer, or other step-count monitoring interventions. Pedometers and other step-count monitoring interventions include all devices, either physical or electronic, which measure the user's step-count, and thus this include pedometers, mobile phone applications and common body-worn fitness devices such as a Fitbit. - Community-based intervention programmes, including primary care. | - Studies which do not measure physical activity as an outcome component within the study, using objective measures. - Other physiological or psychological measurements, those that are not known objective measurements of physical activity. - The use of pedometers and other step-count monitoring interventions in confined simulation environments, including a laboratory. - Interventions taking place in an acute secondary or tertiary care setting, which is not representative of a community-based environment. |
| C | Comparator | - Not used either a pedometer, or other step-count monitoring interventions. - Predominantly received ‘usual standard care’ or healthcare advice with minimal active engagement, at the time the eligible study was performed. |  |
| O | Outcome | - Studies which provided objectively measure outcomes in the form of change in step-count at follow-up compared to baseline were included. - All follow-up periods, either immediate, short-term, medium-term or long-term, following the completion of the intervention, were considered. | - Subjective measures or self-reported measures of step-counts were excluded. |
| S | Study Design | - Randomised Controlled Trials (RCTs) - Published since 1^st^ January 2000. - Published in English. | - Non-randomised controlled trials. |
