# Supplementary material for: The effects of step-count monitoring interventions on physical activity: systematic review and meta-analysis of community-based randomised controlled trials in adults
Source: Int J Behav Nutr Phys Act. 2020 Oct 9;17:129. doi: 10.1186/s12966-020-01020-8 (PMC7545847; doi:10.1186/s12966-020-01020-8)
Supplement: Supplementary file 4 — Additional file 4. Title/Abstract Screening Reviewer Checklist Eligibility. [file 12966_2020_1020_MOESM4_ESM.docx]

**Additional File 4: Title/Abstract Screening Reviewer Checklist Eligibility**

| Checklist |  |
| --- | --- |
| General Properties |  |
| Aged ≥ 18 |  |
| RCT |  |
| General Community-based Population (exclude specified conditions) |  |
| Condition |  |
| Physical Activity *[does not necessarily need to be a condition or outcome of the study]* |  |
| Intervention(s) / Exposure(s) |  |
| Pedometer or equivalent, or Body-worn Fitness Device, or Mobile Phone Fitness Application |  |
| Objective measures |  |
| Comparator(s) / Control |  |
| Usual standard care (not utilising intervention(s) / exposure(s)) |  |
