# Supplementary material for: The effects of step-count monitoring interventions on physical activity: systematic review and meta-analysis of community-based randomised controlled trials in adults
Source: Int J Behav Nutr Phys Act. 2020 Oct 9;17:129. doi: 10.1186/s12966-020-01020-8 (PMC7545847; doi:10.1186/s12966-020-01020-8)
Supplement: Supplementary file 5 — Additional file 5. Summary of Change-on-Change Studies (32 Studies) and End-point Only Studies (25 Studies) Characteristics. [file 12966_2020_1020_MOESM5_ESM.docx]

**Additional File 5: Summary of Change-on-Change Studies (32 Studies) and End-point Only Studies (25 Studies) Characteristics**

| **Summary of Change-on-Change Studies (32 Studies) Characteristics** | | | | | | | | | | |
| --- | --- | --- | --- | --- | --- | --- | --- | --- | --- | --- |
| **Study First Author and Registration ID** | **Year of Publication** | **Total N** | **Age Range of participants (minimum - maximum)** | **Longest Length of Follow-up (weeks)** | **Setting, Country** | **Inclusion** | **Exclusion** | **Intervention Group Components** | **Control Group Components** | **Funding** |
| **Aguiar** Australian New Zealand Clinical Trials Registry: ACTRN12612000721808 | 2016 | 101 | 20 - 65 | 26 | Hunter region, New South Wales, Australia | 1) male; 2) aged 18-65 years; 3) BMI 25-40 kg/m2; 4) at high risk for developing T2DM (Australian Diabetes risk tool score Z12, self-report) | 1) diagnosed T1DM or T2DM; 2) recent weight loss >5% in the previous 6 months; 3) taking medication that could affect or be affected by lifestyle behaviour changes | PULSE Program: print and video resources on weight loss (Self-Help, Exercise and Diet using Internet Technology [SHED-IT] Weight Loss Program including pedometer), diet modification, and exercise for T2DM prevention  Step-count Monitoring Device: pedometer Outcome Measurement: Yamax Digi-Walker SW-200 | Waitlist | 1) Hunter Medical Research Institute; 2) Lions Club District 201N3 Diabetes Foundation; 3) Aurizon |
| **Ashton** Australian New Zealand Clinical Trials Registry: ACTRN12616000350426 | 2017 | 50 | 18 - 25 | 13 | Hunter region, New South Wales, Australia | 1) age; 2) male; 3) available for assessment sessions; 4) access to email and internet facilities | 1) meeting recommendations for fruit and vegetable intake; 2) meeting PA recommendations; 3) participating in healthy lifestyle program; 4) reported physical or mental health medical condition without GP approval to participate; 5) eating disorder; 6) non-English speaking; 7) disability that precluded participation | HEYMAN: eHealth support (website, wearable device, Facebook support group), face-to-face sessions (group and individual), a personalised food and nutrient report, home-based resistance training equipment and a portion control tool  Step-count Monitoring Device: Jawbone wearable PA tracker and mobile phone UP application Outcome Measurement: Yamax Digi-Walker SW-200 | Waitlist | Hunter Medical Research Institute |
| **Baker** Registration: NS | 2008 | 80 | 18 - 65 | 12 | Glasgow, Scotland, UK | 1) independently ambulatory; 2) English speaking; 3) aged 18–65 years; 4) self-classified as not meeting current PA recommendations | 1) no GP approval to participate for exercise programme | WWW: PA consultation and 12-week pedometer-based walking program  Step-count Monitoring Device: pedometer (Omron HJ-109E Step-O-Meter) Outcome Measurement: Omron HJ-109E Step-O-Meter | Usual care | NS |
| **Biddle** ISRCTN Registry: ISRCTN08434554 | 2015 | 187 | 18 - 40 | 52 | Leicestershire and Northampton-shire, UK | 1) aged 18–40 years; 2) BMI in the obese range or BMI in the overweight range with ≥1 additional risk factor for T2DM | 1) significant medical condition 2) steroid use; 3) T1DM or T2DM; 4) pregnancy; 5) non-English speaking | STAND: 3-hour group-based structured education workshop, use of a self-monitoring tool, and follow-up motivational phone call  Step-count Monitoring Device: Gruve self-monitoring device Outcome Measurement: Actigraph GT3X accelerometer | Information leaflet on T2DM and PA | 1) Medical Research Council (UK) under the National Prevention Research Initiative (Project #91409); 2) NIHR Diet, Lifestyle & Physical Activity Biomedical Research Unit; 3) NIHR Collaboration for Leadership in Applied Health Research and Care – East Midlands; 3) Leicester Clinical Trials Unit |
| **Brakenridge** Australian New Zealand Clinical Trial Registry: ACTRN12614000252617 | 2016 | 153 | 28- 38 | 52 | Sydney and Brisbane, Australia | 1) office workers; 2) working ≥50% full-time equivalent; 3) ambulatory (able to walk at least 10 meters) | 1) pregnancy; 2) allergies to adhesive tape; 3) planned absence from work for >2 weeks during study period; 4) own activity-permissive workstation at baseline assessment | Group ORG + tracker: organisational support for increasing PA through regular e-mails and activity tracker  Step-count Monitoring Device: LUMOback activity tracker Outcome Measurement: ActivPAL3 monitor | Organisational support only | 1) Vanguard Grant (Award ID: 100216) from the National Heart Foundation of Australia; 2) Office Ergonomics Research Committee Grant |
| **Davies** ISRCTN Registry: ISRCTN80605705 | 2016 | 880 | 25 - 75 | 156 | Leicestershire, UK | 1) at risk of T2DM using Leicester Diabetes Practice Risk Score and the top 10% were invited; 2) aged 40-75 years if White European, or 25–75 years if South Asian; 3) oral glucose tolerance test demonstrating pre-diabetes | 1) no informed consent; 2) pregnant or lactating; 3) had established T2DM or terminal illness; 4) non-English speaking, requiring interpreter (otherwise South Asian languages accommodated within this study) | Let's Prevent Diabetes: 6-hour group structured education programme including pedometer with refresher course, and regular phone contact  Step-count Monitoring Device: pedometer Outcome Measurement: New Lifestyles NL-800 | Standard care | NIHR Programme Grants for Applied Research scheme (RP-PG-0606-1272) |
| **Finkelstein** ClinicalTrials.gov Identifier: NCT01855776 | 2016 | 800 | 21 - 65 | 52 | Singapore | 1) informed consent; 2) English-speaking; 3) full-time workers; 4) aged 21–65 years; 5) willing to wear an activity tracker; 6) ambulatory (able to walk ≥ten steps continuously); 7) non-pregnant | 1) non-English/Mandarin/Malay/Tamil speaking or speak major dialects; 2) cognitively impairment or unable to communicate verbally; 3) cannot do 10 minutes of aerobic activities without stopping | TRIPPA: PA educational booklets and Fitbit Zip activity tracker [Fitbit only group], or tracker plus charity incentives [Fitbit & charity group], or tracker plus cash incentives [Fitbit & cash group]  Step-count Monitoring Device: Fitbit Zip wireless activity tracker and access to the Fitbit website Outcome Measurement: ActiGraph triaxial GT-3x+ accelerometer | PA educational booklets | Health Services Research Competitive Research Grant (HSRG/0022/2012) from Ministry of Health, Singapore |
| **Garcia-Ortiz** ClinicalTrials.gov Identifier: NCT02016014 | 2018 | 833 | 20 - 69 | 52 | Spain | 1) aged 20-80 years; 2) informed consent [selected from previous EVIDENT I study] | 1) aged >70 years; 2) unable to exercise; 3) unable to follow Mediterranean diet; 4) selected from the previous EVIDENT I study: with known significant physical or mental health condition in the past 5 years, pregnant women and terminal patients | Evident II: 30-minute PA and dietary counselling session, training to use a smartphone application, and follow-up consultation  Step-count Monitoring Device: smartphone application Outcome Measurement: ActiGraph GT3X accelerometer | PA and dietary counselling | 1) Spanish Ministry of Science and Innovation (MICINN); 2) Carlos III Health Institute/European Regional Development Fund (MICINN, ISCIII/FEDER) (FIS: PI13/00618, PI13/01526, PI13/00058, PI13/01635, PI13/02528, PI12/01474; RETICS: RD12/0005, RD16/0007); 3) Regional Health Management of Castilla y León (GRS 1191/B/15, GRS 909/B/14, GRS 770/B/13); 4) Biomedical Research Institute of Salamanca (IBSAL; IBI16/00008); 5) Infosalud Foundation |
| **Gill** ClinicalTrials.gov Identifier: NCT02413385 | 2019 | 118 | 18 - 85 | 26 | Southwestern Ontario, Canada | 1) aged 18-85 years; 2) ≥1 self-reported or measured risk factors for chronic disease including BMI >25kg/m2, <150min of exercise/week, ≥3 hours sitting/day, consuming <8 fruit or vegetable servings/day, T2DMs; 3) completion of the Physical Activity Readiness-Questionnaire or approval from a health care provider | 1) could not comprehend information and consent form | HealtheSteps™ program (0 to 6 months): lifestyle coaching with Heathesteps app, telephone coaching and private HealtheSteps™ social network  Step-count Monitoring Device: HealtheSteps™ smartphone app Outcome Measurement: Yamax Digi-Walker SW-200 | Usual care and sign-posting to healthy lifestyles resources | 1) Public Health Agency of Canada (Canadian Diabetes Strategy); 2) Sykes Assistance Corporation; 3) Saint Elizabeth Health Care; 4) Dairy Farmers of Canada; 5) Sanofi-Aventis |
| **Glynn** ISRCTN Registry: ISRCTN99944116 | 2014 | 90 | 16 - NS | 8 | Ireland | 1) aged >16 years; 2) active Android smartphone users | 1) did not have an Android smartphone; 2) acute psychiatric illness; 3) pregnant; 4) could not undertake moderate exercise | SMART MOVE: PA educational material and smartphone app  Step-count Monitoring Device: Accupedo-Pro Pedometer app Outcome Measurement: Accupedo-Pro Pedometer app | PA educational material | European Union’s Northern Periphery Programme 2007–2013 [Implementing Transnational Telemedicine Solutions project (reference number: 7.13)] |
| **Grey** ClinicalTrials.gov Identifier: NCT03032731 | 2019 | 60 | 35 - 70 | 12 | Bath, UK | 1) aged 35-74 years; BMI ≥25kg/m2 and <40kg/m2; 3) fluent in English; 4) access to the Internet | 1) diagnosis of: coronary heart disease, chronic kidney disease, T1DM or T2DM, stroke, heart failure, severe hypertension (BP > 180/110 mmHg), peripheral arterial disease or thyroid disorders; 2) currently taking medications that could affect weight; 3) going through menopause; 4) taking part (recent participation in last 2 months) in another lifestyle intervention; 4) undergone a large change in habitual lifestyle or body mass | Evolife: Introductory session, 12weeks’ access to Evolife website and pedometer  Step-count Monitoring Device: pedometer Outcome Measurement: BodyMedia SenseWear ‘Core’ monitors | Introductory session and NHS online health resources | Evolution Education Trust |
| **Harris** ISRCTN Registry: ISRCTN42122561 | 2015 | 298 | 60 - 75 | 52 | Oxfordshire and Berkshire, UK | 1) ambulatory outside; 2) no contra-indications to increasing physical activity | 1) residential or nursing home residents; 2) having a physical or mental health condition unsuitable for the intervention | PACE-Lift: primary care nurse PA consultations over 3 months, pedometer step-count and accelerometer PA intensity feedback, and individual PA diary and plan  Step-count Monitoring Device: pedometer (Yamax Digi-Walker SW-200) Outcome Measurement: Actigraph GT3X+ accelerometer | Usual care | NIHR Research for Patient Benefit programme (PB-PG-0909- 20055) |
| **Harris** ISRCTN Registry: ISRCTN98538934 | 2017 | 1023 | 45 - 75 | 52 | South London, UK | 1) aged 45-75 years; 2) no contraindications to increasing MVPA | 1) care-home residents; 2) those with unsuitable conditions | PACE-UP: pedometers, 12-week walking programmes, PA diaries [postal], or 3 PA consultations [nurse-led]  Step-count Monitoring Device: pedometer (Yamax Digi-Walker SW-200) Outcome Measurement: Actigraph GT3X+ accelerometer | Usual care | NIHR Health Technology Assessment Programme (HTA 10/32/02 ISRCTN42122561) |
| **Harris** ISRCTN Registry: ISRCTN42122561 and ISRCTN98538934 | 2018 | 1321 | 45 - 75 | 208 | Oxfordshire, Berkshire and South London, UK | PACE-Lift: 1) ambulatory outside; 2) no contra-indications to increasing physical activity PACE-UP: 1) aged 45-75 years; 2) no contraindications to increasing MVPA | PACE-Lift: 1) residential or nursing home residents; 2) having a physical or mental health condition unsuitable for the intervention PACE-UP: 1) care-home residents; 2) those with unsuitable conditions | PACE-Lift: primary care nurse PA consultations over 3 months, pedometer step-count and accelerometer PA intensity feedback, and individual PA diary and plan PACE-UP: pedometers, 12-week walking programmes, PA diaries [postal group], or 3 PA consultations [nurse-led group]  Step-count Monitoring Device: pedometer (Yamax Digi-Walker SW-200) Outcome Measurement: Actigraph GT3X+ accelerometer | Usual care | 1) Collaboration for Leadership in Applied Health Research and Care South London; 2) NIHR Research for Patient Benefit programme (PB-PG-0909- 20055); 3) NIHR Health Technology Assessment Programme (HTA 10/32/02 ISRCTN42122561) |
| **Ismail** ISRCTN Registry: ISRCTN84864870 | 2019 | 1742 | 40 - 74 | 104 | South London, UK | 1) aged 40–74 years; 2) high CVD risk; 3) not known to have cardiovascular disease or to be on diabetes mellitus, kidney, atrial fibrillation or stroke registers; 4) cardiovascular disease risk score of ≥20%; 5) fluent in conversational English; 6) permanent residency and planning to stay in the UK for ≥3/4 of the year | 1) established cardiovascular disease; 2) pacemaker; 3) being on a register for diabetes mellitus, kidney disease, atrial fibrillation or stroke; 4) chronic obstructive pulmonary disease; 5) disabling neurological disorder; 6) severe mental illness; 7) registered blind; 8) housebound or nursing home resident; 9) not being ambulatory; 10) >3 falls in previous year; 11) pregnancy; 12) advanced cancer; 12) morbid obesity (BMI ≥50kg/m2); 13) currently participating in a weight-loss programme | Move It: enhanced motivational interviewing in 2 formats [individual or group] consisting of 10 sessions, a pedometer alongside training manual, intervention curriculum and participant workbook  Step-count Monitoring Device: pedometer Outcome Measurement: ActiGraph GT3X accelerometer | Local NHS Health Check pathway for cardiovascular disease risk score of ≥20% | 1) NIHR Health Technology Assessment programme (Project: 10/62/03); 2) NIHR Mental Health Biomedical Research Centre; 3) NIHR Health Education England Senior Lectureship Award |
| **Mackey** ClinicalTrials.gov Identiﬁer: NCT0252765 | 2019 | 58 | 61 - 88 | 12 | Vancouver, British Columbia, Canada | 1) community-dwelling men; 2) aged ≥60 years; 3) wanted to be more physically active; 4) no participation in any strength training or aerobic exercise classes in the past 3 months; 5) did not have plans to be out of town for ≥7days during the study; 6) written permission from physician if answered “Yes” to the Physical Activity Readiness Questionnaire Plus | NS | Men on the Move: trained activity coaches delivered participant PA consultations, group-based motivational meetings, weekly telephone support, recreational passes, pedometers and diaries for self-monitoring  Step-count Monitoring Device: pedometer (Omron HJ-112) Outcome Measurement: Actigraph GT3X+ accelerometer | Waitlist | 1) Team Grant in Boys’ and Men’s Health from the Canadian Institutes of Health Research (138295); 2) Scholar Award from the Michael Smith Foundation for Health Research; 3) New Investigator Award from the Canadian Institutes of Health Research |
| **Mann** ClinicalTrials.gov Identifier: NCT01473654 | 2016 | 54 | 18 - NS | 26 | New York City, USA | 1) aged >18 years; 2) English-speaking; 3) diagnosis of prediabetes with HbA1c of 5.7–6.4% or fasting glucose of 100–125 mg/dL | 1) diagnosis of T1DM or T2DM or ever been prescribed a diabetic medication; 2) non-ambulatory; 3) no access to email | ADAPT: healthcare professional consultations and action planning tool with pedometer  Step-count Monitoring Device: pedometer (Omron HJ-720ITC) Outcome Measurement: Omron HJ-720ITC | PA educational material on pre-diabetes | National Institute for Diabetes, Digestive and Kidney Diseases (5K23DK081665) |
| **Mansi** Australian New Zealand Clinical Trials Registry: ACTRN12613000087752 | 2015 | 58 | 18 - 65 | 24 | South Island, New Zealand | 1) not regularly physically active (<7,500 steps/day); 2) ambulatory for ≥10 minutes; 3) able to read and sign an informed consent form and questionnaires; 4) willing to participate for study duration | NS | Pedometer to self-monitor their PA, whilst undertaking a brief intervention, goal setting and PA educational material  Step-count Monitoring Device: pedometer (Yamax Digi-Walker SW-200) Outcome Measurement: Yamax Digi-Walker SW-200 | PA educational material | 1) Mark Steptoe Memorial Trust; 2) School of Physiotherapy; 3) University of Otago |
| **Martin** ClinicalTrials.gov Identifier: NCT01917812 | 2015 | 48 | 18 - 69 | 2 | Baltimore, Maryland, USA | 1) aged 18-69 years; 2) using a Fitbug-compatible smartphone | NS | Phase I: Fitbug Orb and smart-text feedback and goal-setting  Step-count Monitoring Device: Fitbug Orb Outcome Measurement: Fitbug Orb | Blinded | 1) PJ Schafer Cardiovascular Research Fund (unrestricted grant); 2) National Institutes of Health training grant (T32HL07024); 3) Pollin Cardiovascular Prevention Fellowship; 4) Marie-Josee and Henry R Kravis Endowed Fellowship; 5) Howard C. Silverman prize; 6) American Heart Association’s Council on Lifestyle and Cardiometabolic Health; 7) Steven N. Blair Award for Excellence in Physical Activity Research; 8) Aetna Foundation; 9) Kenneth Jay Pollin Professorship in Cardiology |
| **Morgan** Australian New Zealand Clinical Trials Registry: ACTRN12610000699066 | 2012 | 159 | 18 - 65 | 26 | Hunter region, New South Wales, Australia | 1) overweight and obese men (BMI between 25 and 40 kg/m2); 2) access to a mobile phone and a computer with email and Internet facilities | 1) history of major medical problems such as heart disease in the preceding 5 years; 2) diabetes and orthopaedic or joint problems that would be a barrier to walking; 3) recent weight loss of 5 % or more; 4) taking medications that might affect body weight | SHED-IT: weight loss materials, including DVD, handbooks, pedometer, tape measure [resources group], or materials plus study website and efeedback [resources & online group]  Step-count Monitoring Device: pedometer (Yamax Digi-Walker SW-200) Outcome Measurement: Yamax Digi-Walker SW-200 | Waitlist | 1) Heart Foundation Grant-in-Aid; 2) Senior Research Fellowship from the National Health and Medical Research Council of Australia; 3) Career Development Award from the National Health and Medical Research Council of Australia |
| **Mutrie** ISRCTN Registry: ISRCTN70658148 | 2012 | 41 | 65 - 83 | 12 | Glasgow, Scotland, UK | 1) living independently; 2) not meeting PA recommendations | 1) refusals; 2) non-ambulatory outside independently; 3) unable to understand the rationale behind the trial; 4) no GP approval for medical reasons | PA consultations, pedometer and a walking programme  Step-count Monitoring Device: pedometer (New Lifestyles NL-1000) Outcome Measurement: ActivPAL monitor | Waitlist | 1) Chief Scientist Office [CSO] Scotland (CZH/4/457); 2) NHS Research and Development from Greater Glasgow and Clyde; 3) Scottish Primary Care Research Network |
| **Nanda** ClinicalTrials.gov Identiﬁer: NCT02794727 | 2019 | 135 | 18 - 65 | 12 | Rochester, Minnesota, USA | 1) aged 18-65 age; 2) employed full-time; 3) not used a wearable activity tracker within 2 weeks of study entry; 3) agreed not to use any other activity tracker; 4) stable weight (<10% weight change in 3 months); 5) not pregnant; 6) no history of joint problems; 7) ability to participate fully in all aspects of the study; 8) no history of condition that would preclude study participation, hinder study adherence, or skew data collection as judged by the clinical investigator | NS | Activity tracker, feedback sessions and goal-setting   Step-count Monitoring Device: Fitbit Outcome Measurement: Fitbit | Blinded | 1) Mayo Clinic Department of Medicine; 2) staff support through the Department of Medicine Clinical Research Office; 3) REDCap (data entry system used) was supported by Center for Clinical and Translational Science (UL1 TR000135) from the National Center for Advancing Translational Sciences |
| **Oliveira** Australian New Zealand Clinical Trials Registry: ACTRN12614000016639 | 2019 | 131 | 60 - NS | 52 | Sydney, Australia | 1) community-dwelling; 2) aged ≥ 60 years; 3) living at home; 4) regular weekly users of the Internet via a computer or tablet device; 5) regularly able to leave the house without physical assistance from another person | 1) housebound; 2) cognitive impairment (diagnosis of dementia or a Memory Impairment Screen score of < 5); 3) insufficient English language skills to fully participate in the program; 4) progressive neurological disease; 5) medical condition precluding exercise; 6) already meeting Australian Physical Activity Guidelines for older adults (150 minutes of moderate-intensity physical activity per week); 7) falls risk assessment in the past year | Physiotherapist visit, fortnightly telephone-based health coaching, a pedometer, tailored fall prevention advice, and a fall prevention brochure  Step-count Monitoring Device: pedometer (Fitbit tracker) Outcome Measurement: GT3X-BT accelerometer | Usual care and fall prevention brochure | 1) Marrickville Council Community Grant; 2) New South Wales Office of Communities, Sport and Recreation Participation and Facility Program; 3) Coordenação de Aperfeiçoamento de Pessoal de Nível Superior; 4) Research Fellowship from the National Health and Medical Research Council; 5) Career Development Fellowship from the National Health and Medical Research Council |
| **Poirier** ClinicalTrials.gov Identifier: NCT02229409 | 2016 | 265 | 18 - NS | 6 | Franklin, Tennessee, USA | 1) can log on to a password­protected website to provide informed consent; 2) answer a screening questionnaire | 1) failure to complete registration; 2) prior use of Walkadoo; 3) self­-reported limited physical mobility; 4) projected lack of Internet access for ≥4days during study period; 5) insufficient activity tracker wear during the run­-in period | Activity tracker, Internet-based walking program and adaptive goal-setting   Step-count Monitoring Device: Pebble+ wireless accelerometer  Outcome Measurement: Pebble+ wireless accelerometer | Usual care | MeYou Health LLC |
| **Recio-Rodriguez** ClinicalTrials.gov Identifier: NCT02016014 | 2016 | 833 | 20 - 69 | 13 | Spain | 1) aged 20-80 years; 2) informed consent [selected from previous EVIDENT I study] | 1) aged >70 years; 2) unable to exercise; 3) unable to follow Mediterranean diet; 4) selected from the previous EVIDENT I study: with known significant physical or mental health condition in the past 5 years, pregnant women and terminal patients | Evident II: 30-minute PA and dietary counselling session, training to use a smartphone application, and follow-up consultation  Step-count Monitoring Device: smartphone application Outcome Measurement: ActiGraph GT3X accelerometer | PA and dietary counselling | 1) Spanish Ministry of Science and Innovation (MICINN); 2) Carlos III Health Institute/European Regional Development Fund (MICINN, ISCIII/FEDER) (FIS: PI13/00618, PI13/01526, PI13/00058, PI13/01635, PI13/02528, PI12/01474; RETICS: RD12/0005, RD16/0007); 3) Regional Health Management of Castilla y León (GRS 1191/B/15, GRS 909/B/14, GRS 770/B/13); 4) Biomedical Research Institute of Salamanca (IBSAL; IBI16/00008); 5) Infosalud Foundation |
| **Renaud** ClinicalTrials.gov Identiﬁer: NCT03115645 | 2020 | 244 | 18 - NS | 35 | Netherlands | 1) a permanent or a temporary employment contract lasting for at least the 12-month duration of the study at a large insurance company in the Netherlands | 1) contract <0.6 full time equivalents; 2) condition that could affect adherence to the intervention protocol (for example, being wheelchair bound); 3) pregnant | Dynamic Work: environmental components (that is, sit-stand workstations), organisational components (that is, group sessions), and individual components (for example, activity/sitting trackers)  Step-count Monitoring Device: Activator Outcome Measurement: activPAL micro | Usual care including a physically active lifestyle at work | Achmea, Interne Diensten N.V. |
| **Ribeiro** ClinicalTrials.gov Identifier: NCT01567007 | 2014 | 195 | 40 - 50 | 26 | Sao Paulo, Brazil | 1) middle-age female; 2) physically inactive during their leisure time (<30 minutes) from self-reported questionnaire; 3) aged 40-50 years | 1) medication for weight reduction; 2) BMI ≥40; 3) uncontrolled hypertension; 4) T1DM or T2DM; 5) functional limitations preventing them from walking | PA educational sessions through individual counselling [individual counselling group) or group counseling [group counselling]  Step-count Monitoring Device: pedometer (Yamax Digi-Walker SW-200) Outcome Measurement: Digiwalker, Power Walker Model, PW 610 | PA educational sessions and material | 1) Fundacao de Amparo a Pesquisa do Estado de Sao Paulo; 2) Conselho Nacional de Pesquisa |
| **Tudor-Locke** ClinicalTrials.gov Identiﬁer: NCT01519583 | 2020 | 120 | 45-75 | 13 | Louisiana, USA | 1) non-exercising (<7500 steps per day); 2) BMI 20–45 kg/m2 or waist circumference >88 cm; 3) postmenopausal women; 4) aged 45-75 years; 5) Blood pressure of systolic <180 mmHg and diastolic <100 mmHg | 1) signiﬁcant cardiovascular disease; 2) disorders or other signiﬁcant medical conditions (not excluding T2DM) | Walk More: walk at least 10,000 steps/ day [basic group] or 10,000 steps/day and ≥30 minutes in moderate intensity (that is, at a cadence of at least 100 steps/minute) [enhanced group]  Step-count Monitoring Device: pedometer (New Lifestyles NL-1000) Outcome Measurement: Actigraph GT3X+ accelerometer | Usual care | 1) award from American Heart Association; 2) National Institutes of Health (P30 DK072476 and U54 GM104940) |
| **Unick** ClinicalTrials.gov Identiﬁer: NCT01183689 | 2017 | 599 | 18 - 35 | 104 | North Carolina and Rhode Island, USA | 1) normal weight (BMI: 21 to <25 kg/m2) or overweight (BMI: 25-30 kg/m2); 2) aged 18-35; 3) English speaking; 4) no medical conditions that would limit ability to make dietary or PA changes; 5) required to pass screening and baseline assessment visits | 1) 10 pound weight loss in the past six months; 2) bariatric surgery; 3) hospitalization for depression or psychiatric disorder; 4) history of bipolar disorder, manic depression or schizophrenia; 5) past diagnosis or treatment for anorexia or bulimia nervosa; 6) past diagnosis or current symptoms of alcohol or substance abuse; 7) currently pregnant or nursing within the past 6 months or planning to become pregnant within the next 6 months; 8) untreated hypertension, hyperlipidaemia, or T2DM, unless permission is provided by their health care provider; 9) unable to walk for physical activity; 10) the following health problems: heart condition, chest pain during periods of activity or rest, loss of consciousness, diabetes treated with insulin or medications that may cause hypoglycaemia, active tuberculosis, HIV, acromegaly, Cushing’s syndrome, chronic hepatitis B or C, inflammatory bowel disease requiring treatment within the past year, thyroid disease, renal disease, liver disease, hospitalization for asthma in the past year, or cancer within the past 5 years (except for non-melanoma skin cancers or early stage cervical cancer) or chronic use of steroid medication; 11) participation in another weight loss or physical activity study; 12) another member of the household is a participant or staff member on this trial; 13) reason to suspect that the participant would not adhere to the study intervention or assessment schedule; 14) residence or place of work >50 miles from the intervention site; 15) perceived inability to attend the 2 year data collection visit; 16) no Internet access on a regular basis | SNAP: reduce calorie intake by 100 kcals/day & add 2000 steps/day [small change group] alongside group-based session sand self-regulation with pedometers  Step-count Monitoring Device: pedometer Outcome Measurement: Sensewear Armband | Self-guided with one group session on weight gain information, newsletters and electronic resources | National Heart, Lung, and Blood Institute and National Institutes of Health (U01HL090864 and U01HL090875) |
| **Wyke** ISRCTN Registry: ISRCTN-81935608 | 2019 | 1113 | 30 - 65 | 52 | England, Netherlands, Norway and Portugal | 1) male; 2) aged 30–65 years; 3) self-reported BMI of≥27 kg/m2; 4) consented to study procedures | 1) contraindication to moderate intensity physical activity in the Physical Activity Readiness Questionnaire-Plus questionnaire; 2) participation in an existing health promotion programme; 3) did not provide at least 4 days of usable activity monitor data at baseline | EuroFIT: group-based programme delivered by weekly coaches to improve physical activity, sedentary time, and diet using a pocket-worn device (SitFIT) to allow self-monitoring, and a game-based app (MatchFIT) to encourage between-session social support  Step-count Monitoring Device: SitFIT device Outcome Measurement: activPAL monitor | Waitlist | 1) European Union’s Seventh Framework Program (602170); 2) Chief Scientist Office of the Scottish Government Health Directorates |
| **Yates** ClinicalTrials.gov Identifier: NCT00566319 | 2009 | 87 | 18 - NS | 56 | Leicester, UK | 1) overweight or obese individuals (BMI ≥25 or ≥23 kg/m2 for South Asians); 2) screening-detected impaired glucose tolerance | 1) taking steroids; 2) diagnosis of T2DM | PREPARE: 3-hour group-based structured education program to promote walking PA using personalized steps/day and pedometers  Step-count Monitoring Device: pedometer (Yamax Digi-Walker SW-200) Outcome Measurement: New Lifestyles NL-800 | Information leaflet on T2DM and PA | Diabetes UK |
| **Yates** ClinicalTrials.gov Identifier: NCT00941954 | 2017 | 808 | 18 - 74 | 156 | Leicestershire, UK | 1) aged 18-74 years; 2) >90th percentile of the calculated risk score (Leicester Practice Risk Score) for T2DM | 1) taking steroids; 2) terminal illness; 3) unable to participate in any walking activity | Walking Away from Type 2 diabetes: 3-hour group-based education programme with pedometer use and refresher sessions  Step-count Monitoring Device: pedometer Outcome Measurement: Actigraph GT3X accelerometer | Information leaflet on T2DM and PA | 1) NIHR Collaboration in Applied Health Research and Care for Leicestershire, Northamptonshire and Rutland; 2) Collaboration for Leadership in Applied Health Research and Care – East Midlands |
| **Summary of End-point Only Studies (25 Studies) Characteristics** | | | | | | | | | | |
| **Study Name and Registration ID** | **Year of Publication** | **Total N** | **Age Range of participants (minimum - maximum)** | **Longest Length of Follow-up (weeks)** | **Setting** | **Inclusion** | **Exclusion** | **Intervention Group Components** | **Control Group Components** | **Funding** |
| **Ashe** Registration: NS | 2015 | 26 | 55 - 70 | 26 | Vancouver, Canada | 1) healthy, community-dwelling women; 2) aged 55–70 years; 3) self-identified as not engaging in strength training or meeting PA recommendations within the last 3 months; 4) able to climb one flight of stairs and walk 400 m | 1) receiving treatment for medical conditions that prevented walking regularly | EASY model lifestyle program: PA group-based education materials and social support, individualized PA prescription, use of a Fitbit activity monitor  Step-count Monitoring Device: Fitbit One Outcome Measurement: ActiGraph GTX3+ accelerometer | Health education sessions | Canadian Institutes of Health Research |
| **Baker** Registration: NS | 2011 | 86 | 18 - 65 | 52 | Glasgow, Scotland, UK | 1) aged 18-65 years; 2) interest in increasing their PA walking levels within a University campus | NS | 4-week walking intervention using email prompts and pedometers  Step-count Monitoring Device: pedometer Outcome Measurement: Omron HJ-104 Step-0-Meter | Usual care | NS |
| **Cheung** Registration: NS | 2012 | 88 | 25 - 54 | 12 | Hong Kong | 1) working adults; 2) aged 25-54 years; 3) not leading physically active lifestyles; 4) possession of a mobile phone | NS | Pedometer only [PED-only group] or pedometers with text messages on behavioral modification prompts and individualized step goals three times per week during working hours [PED-GOAL group] or pedometer plus active lifestyle-based text messages [PED-LYS group]  Step-count Monitoring Device: pedometer (Yamax Digi-Walker SW-700) Outcome Measurement: Pedometer New Lifestyles-2000 | Text messages on stress relief three times per week | Internal Research Grant of The Hong Kong Institute of Education |
| **Compernolle** ClinicalTrials.gov Identifier: NCT02080585 | 2015 | 274 | 18 - 65 | 13 | Belgium | 1) “white-collar” workplaces; 2) aged 18-65 years; 3) Dutch-speaking, employees who had access to the Internet | NS | 1) information booklet on PA; 2) a non-blinded pedometer; 3) computer-tailored step advice  Step-count Monitoring Device: pedometer Outcome Measurement: Omron HJ-203-ED | Waitlist | NS |
| **Croteau** Registration: NS | 2004 | 15 | 68 - NS | 4 | Maine, USA | 1) aged ≥68 years; 2) Mini-Mental State Examination score ≥23; 3) ≤10 score on performance battery test; 4) independent ambulation; 5) not currently enrolled in structured exercise programme | NS | Life Steps: 4-week pedometer-exercise program on older adults’ using counselling and goal-setting  Step-count Monitoring Device: pedometer (Yamax Digi-Walker SW-200) Outcome Measurement: Yamax Digi-Walker SW-200 | Usual care | 1) University of Southern Maine College of Nursing and Health Professions; 2) Payson Funds |
| **Croteau** Registration: NS | 2007 | 147 | 55 - 94 | 24 | Maine and Florida, USA | 1) aged ≥68 years; 2) ambulate independently; 3) able to walk at a speed and/or appropriate gait patterns to allow adequate pedometer readings; 4) wear clothing that allows appropriate pedometer placement; 5) no physician disapproval indicating harmful effects of increased PA | NS | 12-week intervention incorporating counselling, pedometer usage, and self-monitoring  Step-count Monitoring Device: pedometer (Yamax Digi-Walker SW-200) Outcome Measurement: Yamax Digi-Walker SW-200 | Waitlist | College of Nursing and Health Professions at the University of Southern Maine |
| **Hardeman** ISRCTN Registry: ISRCTN72691150 | 2020 | 1007 | 40 - 74 | 13 | East of England, UK | 1) eligible for NHS Health Check; 2) aged 40-74 years; 3) no diagnosis of vascular disease and not on a care pathway for known risk factors (for example, raised blood pressure) | 1) unable to provide written informed consent; 2) General Practitioner considered unsuitable | Step It Up: 5-minute face-to-face discussion, written materials, pedometer, and step chart (alongside NHS Health Check)  Step-count Monitoring Device: pedometer (Yamax Digi-Walker SW-200) Outcome Measurement: Actigraph GT3X+ accelerometer or Actigraph w-GT3X-BT | NHS Health Check only | 1) NIHR Programme Grants for Applied Research programme (Grant Reference Number RP-PG-0608-10079); 2) NIHR Clinical Research Network; 3) UK Medical Research Council (MC_UU_12015/3) and NIHR Biomedical Research Centre Cambridge [IS-BRC-1215-20014]; 4) salary support in respect from the NHS in the East of England through Clinical Academic Reserve |
| **Katzmarzyk** ClinicalTrials.gov Identifier: NCT01264757 | 2011 | 54 | 35 - 64 | 1 | Louisiana Lower Mississippi Delta region, USA | 1) aged 35-64 years; 2) BMI 25-34.9; 3) able to walk without limitation | 1) significant medical health condition including cardiovascular, respiratory, gastrointestinal, neuromuscular, neurological, or psychiatric disorders; 2) musculoskeletal problems interfering with exercise; 3) immunodeficiency problems; 4) malignancies within 5 years; 5) any other medical condition or life-threatening disease that could be aggravated by exercise | 1-week intervention including education materials on PA and pedometer  Step-count Monitoring Device: pedometer (Yamax Digi-Walker SW-200) Outcome Measurement: ActiGraph GT3X accelerometer | Education materials on PA | 1) ARS/USDA Cooperative Agreement (58-6251-8-038); 2) Louisiana Public Facilities Authority |
| **Koizumi** Registration: NS | 2009 | 68 | 60 - 78 | 12 | Iida, Nagano, Japan | 1) physically independent; 2) lived in a small community where the local economy is based on agriculture and forestry | 1) any gait abnormalities that would affect the measurement of daily activity; 2) taking medication for stroke, hypertension, or hormone replacement therapy; 3) diagnosed coronary heart disease; 4) engaging in an established walking program for health benefits | LIFE: 12-week intervention using feedback-based on accelerometer daily PA  Step-count Monitoring Device: Kenz Lifecorder accelerometer Outcome Measurement: Kenz Lifecorder accelerometer | Usual care | NS |
| **Lara** ClinicalTrials.gov Identifier: NCT02136381 | 2016 | 75 | 55 - 70 | 8 | Northeast England, UK | 1) retired in the last two years, or planned to retire in the next two years; 2) access to a personal computer, tablet or smartphone; 3) reliable internet access at home, work and/or via mobile networks | 1) non-English speaking; 2) severe mental health conditions with assessment | LEAP: web-based intervention comprising of lifestyle modules, including self-monitor of steps using a pedometer  Step-count Monitoring Device: pedometer (Omron HJ-203) Outcome Measurement: Axivity AX3 accelerometer | Usual care and sign-posting to relevant guidelines | 1) Lifelong Health and Wellbeing Cross-Council Research Initiative; 2) Partnerships with the UK Health Departments |
| **Lyons** ClinicalTrials.gov Identifier: NCT01869348 | 2017 | 40 | 55 - 79 | 12 | Texas, USA | 1) aged 55-79 years; 2) BMI 25-35; 3) can read and understand English; 4) can read words on a tablet-sized device | 1) self-reported PA >60 minutes/week; 2) health issues preventing safe walking; 3) psychological issues interfering with participation; 4) current use of wearable activity monitor; 5) endorsing cardiovascular risk questions on PA Readiness Questionnaire | Wearable physical activity monitor, tablet device, and telephone counselling  Step-count Monitoring Device: Jawbone Up24 monitor and Jawbone Up app Outcome Measurement: ActivPAL monitor | Waitlist | 1) Claude D Pepper Older Americans Independence Center (P30AG024832); 2) Sealy Center on Aging; 3) Mentored Research Scholar Grant in Applied and Clinical Research (MRSG-14-165-01-CPPB); 4) American Cancer Society; 5)American Heart Association (13BGIA17110021); 6) Cancer Prevention Research Institute of Texas (RP140020) |
| **Malik** Registration: NS | 2015 | 114 | 50 - 80 | 52 | Wisconsin, Milwaukee, USA | 1) ages 50-80 years; 2) averaged ≤8000 steps/day [from Suboc 2014 paper] | 1) BP ≥160/100; 2) physical health conditions including myocardial infarction within 1 month, angina, heart failure or documented left ventricular ejection fraction of ≤45%, renal insufficiency, liver dysfunction, active malignancy, or cognitive impairment; 3) unable to complete one of two treadmill tests [from Suboc 2014 paper] | Pedometer-only intervention [pedometer-only group], or pedometer with an interactive website to increase PA [pedometer & website group]  Step-count Monitoring Device: pedometer (Omron HJ-72OITC) Outcome Measurement: Actigraph GTX3 accelerometer | Usual care | 1) T. Franklin Williams Scholars Award by Atlantic Philanthropies; 2) American Heart Association (10GRNT3880044); 3) John A. Hartford Foundation; 4) Association of Specialty Physicians; 5) National Institute of Health (K23HL089326, HL081587, HL091019); 6) Diabetes Complication Consortium (25732‐1) 6) Doris Duke Foundation; 7) Merck, Sharp, & Dohme Corporation; 8) Veterans Affairs Merit Award (1/01RX000555) [from Suboc 2014 paper] |
| **Miragall** Registration: NS | 2017 | 76 | 18 - 40 | 13 | Valencia, Spain | 1) <30 minutes of moderate-intensity PA three times a week; 2) sedentary or low active (<7500 steps/day for a week); 3) aged 18 -40 years | 1) physical and/or mental health disorder impeding PA; 2) participating in another PA intervention; 3) regular drug or alcohol consumption; 4) other reasons such as not answering the phone, not knowing the hypotheses of study, or declining to participate after meeting criteria | Internet-based motivational intervention supported by pedometers to increase motivation and set individualised PA goals  Step-count Monitoring Device: pedometer (Fitbit One) Outcome Measurement: Fitbit One | Blinded | 1) PROMOSAM Excellence in Research Program (PSI2014-56303-REDT); 2) Center for Physiopathology of Obesity and Nutrition |
| **Nishiguchi** Japan Medical Association Center for Clinical Trials Registry: JMA-IIA00108 | 2015 | 48 | 60 - NS | 12 | Kyoto, Japan | 1) independently community-living individuals; 2) aged ≥60 years; 3) willing to participate in group exercise classes for ≥3 months | 1) history of major psychiatric illness; 2) serious neurological diagnosis; 3) self-reported severe cardiac, pulmonary, or musculoskeletal disorders; 4) cognitive impairment (Mini-Mental State Examination score ≤23; 5) major abnormalities on brain MRI scans, such as cerebral infarction or tumour | Group training, walking exercise assignmnents, pedometer and goal-setting  Step-count Monitoring Device: pedometer (Yamax Power Walker EX-300) Outcome Measurement: Yamax Power Walker EX-300 | Usual care | 1) Grants-in-Aid (25245068) for Scientiﬁc Research from the Japan Society for the Promotion of Science and the Ministry of Education, Culture, Sports, Science and Technology; 2) Uehiro Foundation on Ethics and Education |
| **Ornes** Registration: NS | 2006 | 121 | 18 - 34 | 4 | Cedar City, Utah, USA | 1) aged 18-34 years; 2) not considered physically active; 3) independently ambulatory; 4) follow relevant instructions; 5) comfortable using computer technology | 1) athleteic women at university; 2) majoring in physical education or dance | Theory-based, web-delivered PA intervention on walking behaviour   Step-count Monitoring Device: pedometer (Yamax Digi-Walker SW-200) Outcome Measurement: Yamax Digi-Walker SW-200 | Blinded waitlist | 1) Southern Utah University; 2) Sigma Theta Tau International |
| **Pal** Australian New Zealand Clinical Trials Registry: ACTRN12609000176268 | 2011 | 28 | 35 - 55 | 12 | Perth, Australia | 1) aged 35-55 years; 2) sedentary, overweight and obese women (BMI >25 and <35 kg/m2) | 1) physical and mental health including renal failure, hypothyroidism, T1DM/T2DM, heart conditions or gastrointestinal surgery; 2) pregnancy, lactating or planning pregnancy; 4) smoking; 5) >2 hours of moderate intensity PA/week | Accumulate 10,000 steps/day using pedometers  Step-count Monitoring Device: pedometer (Yamax Digi-Walker SW-200) Outcome Measurement: Yamax Digi-Walker SW-200 | Blinded, advised to achieve 3,000 steps/day | Healthway |
| **Pears** ISRCTN Registry: ISRCTN02863077 | 2016 | 394 | 40 - 74 | 4 | East of England, UK | 1) aged 40–74 years; 2) not previously diagnosed with heart disease, stroke, diabetes, or kidney disease | 1) non-English speaking | Very brief interventions for PA using pedometer [pedometer only group] or combined with motivational inteviewing [pedometer & motivational group]  Step-count Monitoring Device: pedometer (Yamax Digi-Walker SW-200) Outcome Measurement: ActiGraph GT3X+ accelerometer | Usual care | 1) NIHR Programme Grants for Applied Research Programme (RP-PG-0608-10079); 2) NIHR Biomedical Research Centre at Guy’s and St Thomas’ NHS Foundation Trust and King’s College London |
| **Rowley** Registration: NS | 2017 | 170 | 55 - 80 | 12 | Milwaukee, USA | 1) aged 55-80 years; 2) inactive or insufficiently active (<7,500steps/day); 3) no orthopaedic limitations to walking; 4) expressed interest in starting a walking program; 5) access to a computer and Internet | NS | Website-mediated education, goal-setting with pedometer, self-regulation and feedback (internet and pedometer group) or goal-setting with pedometer only (pedometer-only)  Step-count Monitoring Device: pedometer (Omron HJ-720ITC) Outcome Measurement: Omron HJ-720ITC | Usual care | National Institute on Aging (5K01AG025962) |
| **Simons** ClinicalTrials.gov Identiﬁer: NCT0294880 | 2018 | 130 | 18 - 30 | 22 | Flanders, Belgium | 1) employed; 2) aged 18-30 years; 3) lower educated (no university or college degree); 4) not meeting the physical activity guidelines of 150 minutes of MVPA/week; 5) not using an activity tracker; 6) not participating in a sports program (via a website, an app, or a sports center); 7) in possession of an Android smartphone | NS | Active Coach app: with Fitbit activity tracker to allow personal goals, practical tips, and educational facts for PA  Step-count Monitoring Device: Fitbit Charge Outcome Measurement: Actigraph GT3X+ accelerometer | Print-based generic PA information | 1) PhD fellowship of The Research Foundation Flanders (11U8114N); 2) The Research Foundation Flanders (FWO; postdoctoral research fellowship: FWO11/PDO/097); 3) National Heart Foundation of Australia Future Leader Fellowship (ID 100427) |
| **Suboc** ClinicalTrials.gov Identifier: NCT0121297 | 2014 | 102 | 50 - 80 | 12 | Wisconsin, Milwaukee, USA | 1) aged 50-80 years; 2) averaged ≤8000 steps/day | 1) BP ≥160/100; 2) physical health conditions including myocardial infarction within 1 month, angina, heart failure or documented left ventricular ejection fraction of ≤45%, renal insufficiency, liver dysfunction, active malignancy, or cognitive impairment | Pedometer-only intervention [pedometer-only group], or pedometer with an interactive website to increase PA [pedometer & website group]  Step-count Monitoring Device: pedometer (Omron HJ-72OITC) Outcome Measurement: Actigraph GTX3 accelerometer | Usual care | 1) T. Franklin Williams Scholars Award by Atlantic Philanthropies; 2) American Heart Association (10GRNT3880044); 3) John A. Hartford Foundation; 4) Association of Specialty Physicians; 5) National Institute of Health (K23HL089326, HL081587, HL091019, T32HL007792); 6) Doris Duke Foundation; 7) Merck, Sharp, & Dohme Corporation; 8) Veterans Affairs Merit Award (1/01RX000555) |
| **Takahashi** Registration: NS | 2016 | 130 | 18 - NS | 8 | Rochester, Minnesota, USA | 1) aged ≥18 years; 2) community dwelling; 3) overweight or obese; 4) multiple medical comorbid conditions; 5) extended and complex tiering in determining medical complexity | 1) ≤ tier 2 for determining complexity; 2) clinical dementia; 3) wheelchair-bound; 4) uncontrolled depression (Patient Health Questionnaire-9 score >10); 5) in a hospice, skilled nursing facility or in a correctional facility; 8) declined medical record review | Pedometer use with goal setting using consultations and PA educational material  Step-count Monitoring Device: pedometer (Omron HJ-112) Outcome Measurement: Omron HJ-11 | Waitlist | National Center for Advancing Translational Sciences (UL1 TR000135) |
| **Talbot** Registration: NS | 2011 | 156 | 18 - NS | 24 | Maryland and Washington DC, USA | 1) part-time guard members; 2) had failed the 2-mile run component of the Army Physical Fitness Test; 3) ≥12 months before re-enlistment or retirement | 1) history of coronary heart disease or stroke; 2) on hypertensive or cholesterol-lowering medications; 3) pregnancy <6 months; 4) post-menopausal or on hormone replacement therapy; 5) major musculo-skeletal disorders | Fitness for Life: pedometer-based behavioural intervention using counselling sessions to promote PA  Step-count Monitoring Device: pedometer (Yamax Digi-Walker SW-200) Outcome Measurement: Yamax Digi-Walker SW-200 | Usual fitness guidance | 1) Johns Hopkins Bayview General Clinical Research Center (Department of Health and Human Services, National Institutes of Health, National Center for Research Resources (M01 RR0279)); 2) Intramural Research Program of the National Institute on Aging; 3) Triservice Nursing Research Program of Johns Hopkins Bayview General Clinical Research Center |
| **Thorndike** ClinicalTrials.gov Identiﬁer: NCT01287208 | 2014 | 104 | 23 - 37 | 6 | Boston, Massachusetts, USA | 1) medicine residents | 1) children | Be Fit: activity monitor and access to exercise and nutrition program  Step-count Monitoring Device: Fitbit Outcome Measurement: Fitbit | Blinded and access to exercise and nutrition program | National Institutes of Health/National Heart Lung and Blood Institute (K23 HL93221) |
| **Warren** ISRCTN Registry: ISRCTN73725618 | 2014 | 131 | 40 - 74 | 12 | Devon, Bristol and Coventry, UK | 1) aged 40-74 years; 2) classified as inactive using PA questionnaire; 3) able to walk continuously for 5 minutes without fatigue or discomfort | NS | Written and verbal brief GP advice and pedometer use to self-monitor PA  Step-count Monitoring Device: pedometer (New Lifestyles NL-800) Outcome Measurement: New Lifestyles NL-800 | Written PA advice | Medical Research Council (G0802118/1) |
| **Yamada** Registration: NS | 2012 | 87 | 65 - NS | 26 | Japan | 1) aged ≥65 years; 2) walk <5,000 steps/day; 3) community-dwelling; 4) visited a primary care physician within 3 years; 5) no severe cognitive impairment using screening test; 6) could walk independently, or with an aid; 7) consent to group exercise classes for ≥6 months; 8) access to transportation; 9) no significant hearing or visual impairments; 10) no regular exercise within 12 months | 1) severe cardiac, pulmonary, or musculoskeletal disorders; 2) co-morbidities associated with greater risk of falls, such as Parkinson's disease and stroke; 3) use of psychotropic drugs | 6-month pedometer-based behavioural change program using goal-setting, self-monitoring, and feedback  Step-count Monitoring Device: pedometer (Yamax Powerwalker EX-510) Outcome Measurement: Yamax Powerwalker EX-510 | Usual care | NS |

Croteau 2007, Mutrie (2012) and Takahashi (2016) had a crossover study design

**Abbreviations for Additional File 5**

NS not stated

UK United Kingdom

USA United States of America

NIHR National Institute for Health Research

T2DM Type 2 Diabetes Mellitus

T1DM Type 2 Diabetes Mellitus

HbA1c glycated haemoglobin

PA physical activity

MVPA moderate-vigorous physical activity

BMI body mass index
