# Supplementary material for: The effects of step-count monitoring interventions on physical activity: systematic review and meta-analysis of community-based randomised controlled trials in adults
Source: Int J Behav Nutr Phys Act. 2020 Oct 9;17:129. doi: 10.1186/s12966-020-01020-8 (PMC7545847; doi:10.1186/s12966-020-01020-8)
Supplement: Supplementary file 6 — Additional file 6. Stata procedure Mvmeta. [file 12966_2020_1020_MOESM6_ESM.docx]

**Additional File 6: Stata procedure Mvmeta**

We used the STATA procedure mvmeta as described in White(2009) and White(2011) to fit: (i) a multivariate meat-analysis across different time points; (ii) meta-regressions at the first time point. Specifically we used the updated mvmeta described in White(2011).

*Multivariate meta-analysis at different time points for change-on-change studies only*

The reason for carrying out multi-variate meta-analysis across different time points was that the multiple univariate meta-analyses presented in Figures 3 and 4 ignore the information contained in the estimates from the same study at different time points. Estimates at different time points within the same study are moderately correlated because they are based on the same individuals undergoing similar treatments. By ignoring the correlations we are ignoring information and losing power, particularly if we have a time point with few data points as at 2 years.

We estimated the relevant within study correlations by using individual level data from PACE-UP and PACE-Lift studies as follows:

Correlations of Steps at time points 9 months to 1 year apart: 0.45

Correlations of Steps at time points >18 months apart: 0.4

We then fitted using:

mvmeta y S, bscov(corr I(5))

The between study covariance matrix was specified as being diagonal. It should be noted that we used the reml method of fitting as the method of moments cannot be used. These multivariate estimates are presented in Table 1.

*Meta-regression at first time point*

We also used mvmeta to fit meta-regressions at the earliest time point (<=3months) and these are presented in Table 2. This was straightforward and simply required the code:

mvmeta y S Design Device G mm2

We used the mm2 option which uses the method of moments to fit the model corresponding with the methods used in the simple meta-analyses in Figures 3 and 4. In fact using mm2 or reml made little difference to the estimates or their confidence limits or statistical significance.

**References**

White IR. Multivariate random-effects meta-analysis. The Stata Journal. 2009;9(1):40-56.

White IR. Multivariate random-effects meta-regression: Updates to mvmeta. The Stata Journal. 2011;11(2):255-270.
